# SMiPoly: Generation of a Synthesizable Polymer Virtual Library Using Rule-Based Polymerization Reactions

**DOI:** 10.1021/acs.jcim.3c00329

**Published:** 2023-08-21

**Authors:** Mitsuru Ohno, Yoshihiro Hayashi, Qi Zhang, Yu Kaneko, Ryo Yoshida

**Affiliations:** † Daicel Corporation, Kita-ku, 530-0011 Osaka, Japan; ‡ The Institute of Statistical Mathematics, Research Organization of Information and Systems, Tachikawa, Tokyo 190-8562, Japan; § The Graduate University for Advanced Studies, SOKENDAI, Tachikawa, Tokyo 190-8562, Japan; ∥ National Institute for Materials Science, 305-0047 Ibaraki, Japan

## Abstract

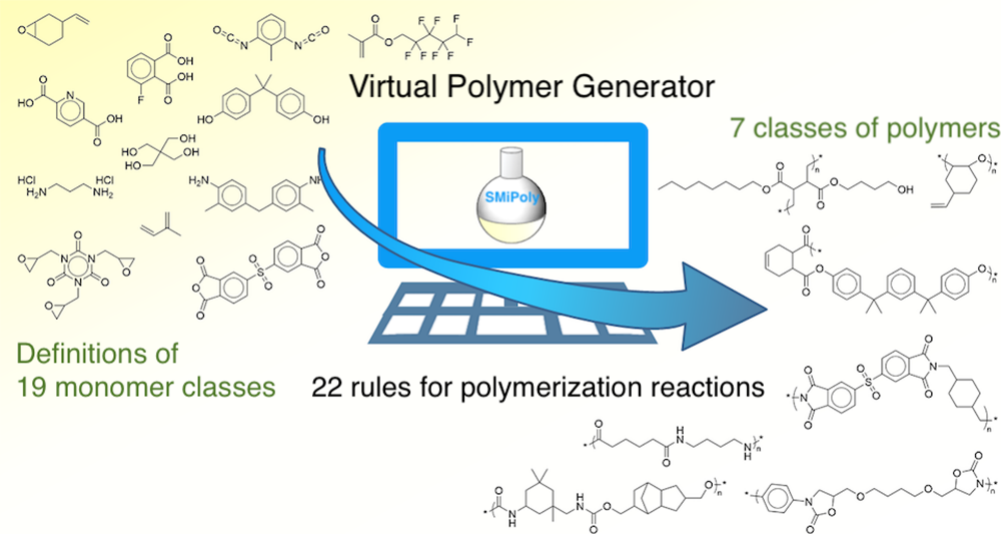

Recent advances in machine learning have led to the rapid adoption of various computational methods for de novo molecular design in polymer research, including high-throughput virtual screening and inverse molecular design. In such workflows, molecular generators play an essential role in creation or sequential modification of candidate polymer structures. Machine learning-assisted molecular design has made great technical progress over the past few years. However, the difficulty of identifying synthetic routes to such designed polymers remains unresolved. To address this technical limitation, we present Small Molecules into Polymers (SMiPoly), a Python library for virtual polymer generation that implements 22 chemical rules for commonly applied polymerization reactions. For given small organic molecules to form a candidate monomer set, the SMiPoly generator conducts possible polymerization reactions to generate an exhaustive list of potentially synthesizable polymers. In this study, using 1083 readily available monomers, we generated 169,347 unique polymers forming seven different molecular types: polyolefin, polyester, polyether, polyamide, polyimide, polyurethane, and polyoxazolidone. By comparing the distribution of the virtually created polymers with approximately 16,000 real polymers synthesized so far, it was found that the coverage and novelty of the SMiPoly-generated polymers can reach 48 and 53%, respectively. Incorporating the SMiPoly library into a molecular design workflow will accelerate the process of de novo polymer synthesis by shortening the step to select synthesizable candidate polymers.

## Introduction

In recent years, data-driven and computer-aided molecular design technologies have spread rapidly^1,2^ across various areas of materials research, such as drug discovery,^3,4^ catalyst design,^5−8^ and polymer design.^9−12^ The task of molecular design boils down to the forward and backward predictions.^13^ The objective of the forward prediction is to obtain a forward mapping from any given chemical structure to its properties. In a conventional scenario, supervised learning is performed to learn the structure–property relationships. In the backward prediction, the inverse mapping of the forward model is obtained to identify promising chemical structures that exhibit desired properties. The most traditional approach to solving the inverse problem is to perform high-throughput virtual screening; millions or billions of candidate molecules are computationally created, and the forward model is used to determine whether their properties fall into the target region or not.^14−16^ Alternatively, adaptive heuristic search algorithms, such as evolutionary computation,^17−19^ Bayesian optimization,^20^ particle swarm optimization,^21^ and Monte Carlo computation^11,13,22^ have also been used to iteratively modify the candidate molecules to achieve the desired properties.

Here, the performance of molecular generators plays a key role in determining the success or failure of molecular design. Traditionally, virtual libraries have been constructed by probabilistically recombining chemical fragments from which substituent groups or ring structures of existing molecules are pre-extracted.^17,23^ Recently, molecular generators using statistically trained generative models have also become widely used.^24^ For example, a probabilistic language model is trained to generate simplified molecular input line entry system (SMILES)^25^ strings by converting existing molecules into the training string set.^11,13,15,22^ Alternatively, deep generative models based on the graph representation of molecules have been studied extensively.^26−28^ While the traditional methods limit the searchable space to a combination of predefined fragment sets, generative machine-learning models can create novel molecules from a wider chemical space. However, it is not guaranteed that such computationally designed virtual molecules are feasible to synthesize.^29^ One way to overcome this drawback is to use synthetic reaction models.^30,31^ From a large set of synthetic reactions of organic compounds in a public database, a deep neural network is trained to predict the synthetic products of a given set of reactants.^32^ In addition to such generative machine-learning models, rule-based models compiling prior knowledge of chemical reactions,^14^ hybrid models of machine learning and rule-based methods,^16,24,33^ and retrosynthetic analysis^34,35^ have been proposed. By feeding commercially available reactants to such a prediction model, a virtual library can be constructed in which synthesizability and synthetic routes are taken into account.

The aforementioned methodologies have been developed primarily for designing small molecules. However, the development of general-purpose polymer generators has lagged far behind that of small molecule generators. Thus far, polymer repeating units with their chemical structures have been generated by statistical generative models trained from existing polymers in the same manner as the generation of small molecules. Wu et al.^11^ designed and synthesized three amorphous polymers with high thermal conductivity by solving an inverse problem using a probabilistic language model trained on synthetic polymers recorded in the polymer properties database PoLyInfo.^36^ In addition, several applications of polymer generative models have been reported for inverse design for the dielectric constant and solubility of polymers.^16,37,38^ Ma and Luo^15^ constructed a virtual library of more than one million polymers using a deep language generative model trained on the synthetic polymers in PoLyInfo as well. Methodological and practical research on the computational design of polymeric materials is less advanced than for small molecules.^39,40^ The lack of data resources for polymeric materials is a significant bottleneck. Furthermore, the synthesis of computationally generated polymers presents a greater technical hurdle than for small materials; in order to estimate the synthesizability of the generated polymers, it is necessary to break a designed polymer down into starting monomers and investigate their availability. Computational methodologies for this have not been well studied.

Recently, Kim et al.^16^ provided a generative model of synthetically accessible polymer repeating units with a rule-based polymerization reaction algorithm. Using this system, they constructed a database called the Open Macromolecular Genome (OMG) that contains highly synthesizable virtual polymers. The OMG will be an important database for data-driven polymer research, but there is room for improvement in the definition of rule sets. From the viewpoint of synthetic organic chemistry, the reactivity of a substrate is influenced by the steric and electrical effects of the substituents in the reaction center. Furthermore, as pointed out in that paper, the selectivity of the reaction is influenced by coexisting functional groups in the reactant molecule. Therefore, it is necessary to provide reaction rules that take these factors into account.

In this study, we built a virtual library generator for polymers that implements a comprehensive rule set for practically applied polymerization reactions with a Python open-source library, Small Molecules into Polymers (SMiPoly). The generators implement 22 reaction rules that consist of six chain polymerization reactions and 16 step-growth polymerization reactions. The types of polymerization reactions used in actual polymer synthesis are quite limited compared to the overall organic synthetic reactions. Our rule set covers a significant portion of common polymerization reactions considered in previous studies.^41−44^

Using 1083 input monomers carefully selected based on availability, cost, safety, and legal compliance, seven classes of polymers, i.e., polyolefins, polyesters, polyethers, polyamides, polyimides, polyurethanes, and polyoxazolidones, were polymerized in silico. The generated virtual polymers have significantly promising synthesizability with the navigation of polymerization routes. The distribution of the 169,347 generated polymers and nearly 16,000 synthesized polymers forming the 21 polymer classes in PoLyInfo were compared to systematically and quantitatively evaluate the coverage and novelty of the virtual library with respect to the existing polymers. Our library generator covered approximately 50% of the chemical space of the polymers synthesized to date, and approximately 50% of the polymers produced were outside the distribution of the existing polymers.

## Methods

**Figure 1:**
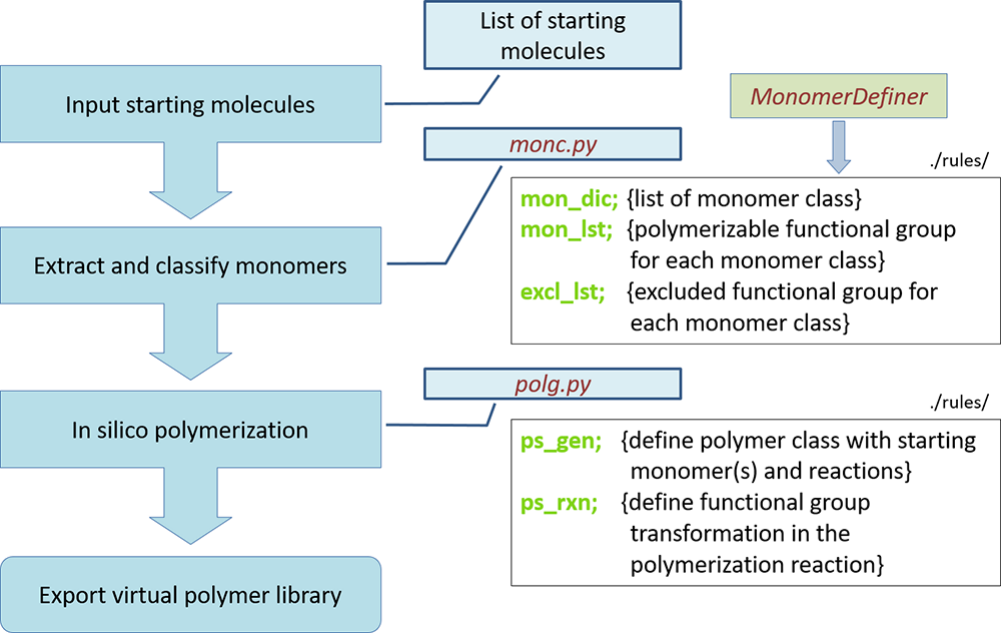
Workflow of the monomer classification and the polymer generation with SMiPoly.

The SMiPoly library implements a rule set of 22 polymerization reactions, which consists of two submodules, “monc.py” and “polg.py” (Figure 1). The submodule “monc.py” extracts monomers from the given set of starting molecules and classifies them into 19 different monomer classes by checking whether or not to include polymerizable functional groups of the 22 polymerization reactions. The submodule “polg.py” applies the functional group transformations programmed into an applicable polymerization reaction to generate the chemical structure of a polymer repeating unit from the given one or two starting monomers. The current implementation is capable of generating seven classes of polymers. In the following, each step is described in detail.

### Polymerization Reactions

**Figure 2:**
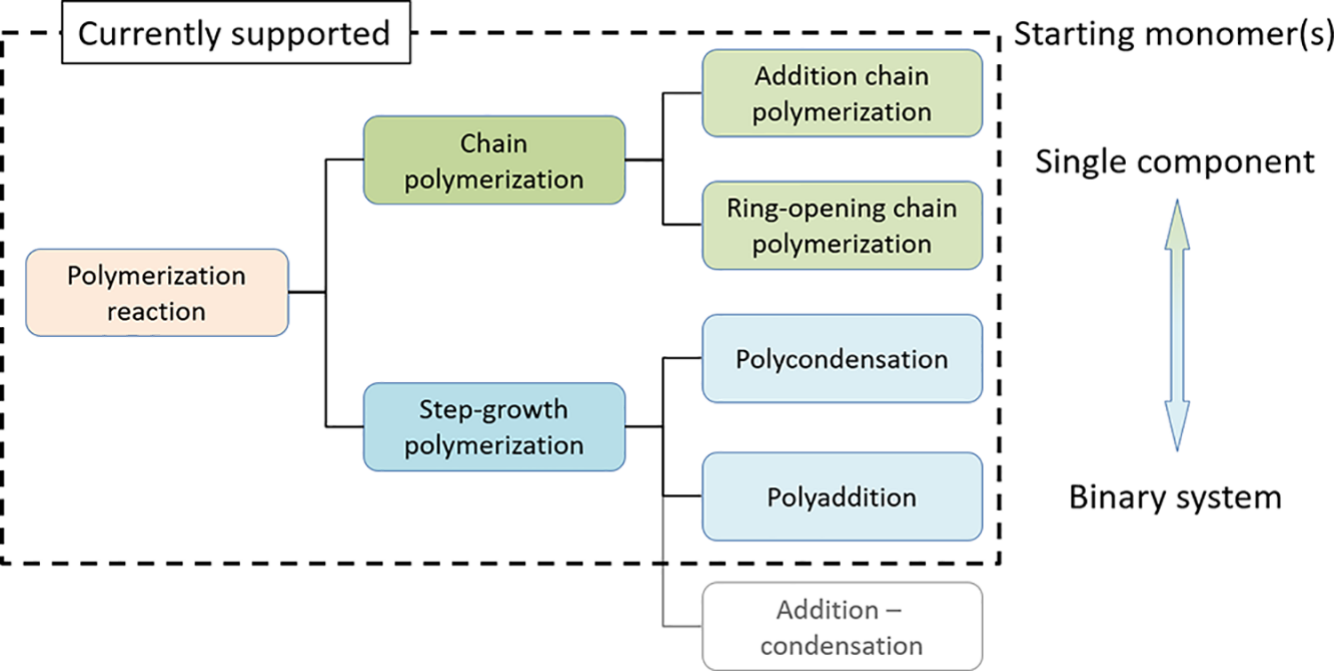
Classification of polymerization reactions implemented in the SMiPoly library. The definition of the terms is in accordance with the chemical terminology of the International Union of Pure and Applied Chemistry (IUPAC)^43,45^ except for “step-growth polymerization” and “addition-condensation”.

The 22 polymerization reactions are classified in accordance with domain knowledge as summarized in Figure 2.^41,42,44^ The definition of the terms follows the chemical terminology of the International Union of Pure and Applied Chemistry (IUPAC)^43,45^ except for “step-growth polymerization” and “addition-condensation” as described below.

At first, we classified common polymerization reactions into two types: chain polymerization and step-growth polymerization. These two types are classified according to the reaction mechanism of the polymer chain growth. The polymerization reactions in which the growth of a polymer chain proceeds exclusively by a chain reaction are categorized as chain polymerization, and others are defined as step-growth polymerization. The step-growth polymerization here indicates a polymerization reaction in which the growth of polymer chains proceeds by reactions between molecules of all degrees of polymerization. The chain polymerization can be further classified into addition chain polymerization and ring-opening chain polymerization on the basis of the type of chemical reactions involved in the growth step. The step-growth polymerization can be classified into three types: polycondensation, polyaddition, and addition-condensation. The addition-condensation here means a polymerization reaction in which addition and condensation reactions are repeated alternately.

Each reaction class consists of several polymerization reactions defined by specific functional group transformations. A polymerization reaction is expressed as a set of the starting monomer(s) and the functional group transformation represented by reaction SMiles ARbitrary Target Specification (SMARTS).^46^ The 22 polymerization reactions currently implemented and their functional group transformations represented by reaction SMARTS are summarized in Table 1, and illustrative examples of the rule-specific functional group transformations are shown in Table ??. The current rule set can generate the seven polymer classes: polyolefin, polyester, polyether, polyamide, polyimide, polyurethane, and polyoxazolidone.

**Table 1:** 22 Polymerization Reactions with Monomer Classes To Be Applied, and Their Functional Group Transformations Represented by Reaction SMARTS^a^

	polymer class	monomer class 1	monomer class 2	reaction type
	polymerization reaction			
1	polyolefin	vinyl		addition chain polymerization
	[CX3;H2,H1,H0:1]=[C;H2,H1,H0:2] ≫*-[C:1][C:2]-*			
2	polyolefin	cyclic olefin		addition chain polymerization
	[CX3;R:1]=[CX3;R:2]≫*-[CX4;R:1][CX4;R:2]-*			
3^b^	polyolefin	vinyl	vinyl	addition chain polymerization
	[ CX3;H2,H1,H0;!R:1]=[CX3;H2,H1,H0;!R:2].[CX3;H2,H1,H0;!R:3]=[CX3;H2,H1,H0;!R:4]≫			
	*-[CX4:1][CX4:2][CX4:3][CX4:4]-*			
4^b^	polyolefin	vinyl	cyclic olefin	addition chain polymerization
	[CX3;H2,H1,H0;!R:1]=[CX3;H2,H1,H0;!R:2].[CX3;H1,H0;R:3]=[CX3;H1,H0;R:4]≫			
	*-[CX4:1][CX4:2][CX4:3][CX4:4]-*			
5^b^	polyolefin	cyclic olefin	cyclic olefin	addition chain polymerization
	[CX3;H1,H0;R:1]=[CX3;H1,H0;R:2].[CX3;H1,H0;R:3]=[CX3;R:4]≫*-[CX4:1][CX4:2][CX4:3][CX4:4]-*			
6	polyester	lactone		ring-opening chain polymerization
	[ CX3;R:1](=[OX1])[OX2;R:2]≫(*-[CX3:1](=[OX1]).[OX2:2]-*)			
7	polyester	hydroxy carboxylic acid^c^		polycondensation
	([OX2H1;!$(OC=*):1].[CX3:2](=[O])[OX2H1])≫(*-[OX2:1].[CX3:2](=[O])-*)			
8^b^	polyester	hydroxy carboxylic acid^c^	hydroxy carboxylic acid^c^	polycondensation
	([OX2H1;!$(OC=*):1].[CX3:2](=[O])[OX2H1]).([OX2H1;!$(OC=*):3].[CX3:4](=[O])[OX2H1])≫			
	(*-[OX2:1].[CX3:2](=[O])[OX2:3].[CX3:4](=[O])-*)			
9	polyester	di/polycarboxylic acid	di/polyol	polycondensation
	([CX3:1](=[O])[OX2H1,Cl,Br].[CX3:2](=[O])[OX2H1,Cl,Br]).([O,S;X2;H1;!$([O,S]C=*):3].[O,S;X2;H1;!$([O,S]C=*):4])≫			
	(*-[CX3:1](=[O]).[CX3:2](=[O])-[O,S;X2;!$([O,S]C=*):3].[O,S;X2;!$([O,S]C=*):4]-*)			
10	polyester^d^	di/polyol	carbon monoxide^e^	polycondensation^f^
	([O,S;X2;H1;!$([O,S]C=*):1].[O,S;X2;H1;!$([O,S]C=*):2]).[C-]#[O+]≫			
	(*-[O,S;X2;!$([O,S]C=*):1].[O,S;X2;!$([O,S]C=*):2][CX3](=[O])-*)			
11	polyester	cyclic anhydride	epoxide	ring-opening chain polymerization
	[C,c;R:1][CX3,c;R](=[OX1])[OX2,o;R][CX3,c;R](=[OX1])[C,c;R:2].[CX4;R:3]1[OX2;R:4][CX4;R:5]1≫			
	([C,c:1][CX3](=[OX1])(-*).[C,c:2][CX3](=[OX1])[OX2][CX4:3][CX4:5][OX2:4]-*)			
12	polyether	epoxide		ring-opening chain polymerization
	[CX4;H2,H1,H0;R:1]1[O;R][C;R:2]1≫*-[CX4:1][CX4:2][O]-*			
13	polyether	hindered phenol		polycondensation^g^
	[c]1([OH1:1])[c:2][c:3][c;H1:4][c:5][c:6]1≫[c]1([OX2:1]-[*])[c:2][c:3][c:4](-*)[c:5][c:6]1			
14	polyether^h^	bis(*p-*halogenated aryl)sulfone	di/polyol (without thiol)	polycondensation
	[c:1]1[c:2]p[c:3]([F,Cl,Br,I])[c:4][c:5][c:6]1[SX4](=[OX1])(=[OX1])[c:7]2[c:8][c:9][c:10]([F,Cl,Br,I])[c:11][c:12]2.			
	([OX2;H1;!$([O,S]C=*):13].[OX2;H1;!$([O,S]C=*):14])≫			
	[c:1]1[c:2][c:3](-[*])[c:4][c:5][c:6]1[SX4](=[OX1])(=[OX1])[c:7]2[c:8][c:9][c:10]([OX2;!$([O,S]C=*):13].			
	[OX2;!$([O,S]C=*):14]-[*])[c:11][c:12]2			
15	polyether^i^	bis(*p-*fluoroaryl)ketone	di/polyol (without thiol)	polycondensation
	[c:1]1[c:2][c:3]([F])[c:4][c:5][c:6]1[CX3](=[OX1])[c:7]2[c:8][c:9][c:10]([F])[c:11][c:12]2.([OX2;H1;!$([O,S]C=*):13].			
	[OX2;H1;!$([O,S]C=*):14])≫			
	[c:1]1[c:2][c:3](-[*])[c:4][c:5][c:6]1[CX3](=[OX1])[c:7]2[c:8][c:9][c:10]([OX2;!$([O,S]C=*):13].			
	[OX2;!$([O,S]C=*):14]-[*])[c:11][c:12]2			
16	polyamide	lactam		ring-opening chain polymerization
	[CX3;R:1](=[OX1])[NX3;R:2]≫(*-[CX3:1](=[OX1]).[NX3:2]-*)			
17	polyamide	amino acid^c^		polycondensation
	([NX3;H2,H1;!$(OC=*):1].[CX3:2](=[O])[OX2H1])≫(*-[NX3:1].[CX3:2](=[O])-*)			
18^b^	polyamide	amino acid^c^	amino acid^c^	polycondensation
	([N&X3;H2,H1;!$(NC=*):1].[CX3:2](=[O])[OX2H1]).([N&X3;H2,H1;!$(NC=*):3].[CX3:4](=[O])[OX2H1])≫			
	(*-[NX3;!$(NC=*):1].[CX3:2](=[O])[NX3;!$(NC=*):3].[CX3:4](=[O])-*)			
19	polyamide	di/polycarboxylic acid	di/polyamine	polycondensation
	([CX3:1](=[O])[OX2H1,Cl,Br].[CX3:2](=[O])[OX2H1,Cl,Br]).([N&X3;H2,H1;!$(NC=*):3].[N&X3;H2,H1;!$(NC=*):4])≫			
	(*-[CX3:1](=[O]).[CX3:2](=[O])-[NX3;!$(NC=*):3].[NX3;!$(NC=*):4]-*)			
20	polyimide	di/polycyclic anhydride	primary di/polyamine	polycondensation
	([CX3,c;R:1](=[OX1])[OX2,o;R][CX3,c;R:2](=[OX1]).[CX3,c;R:3](=[OX1])[OX2,o;R][CX3,c;R:4](=[OX1])).			
	([C,c:5][NX3;H2;!$(N[C,S]=*)].[C,c:6][NX3;H2;!$(N[C,S]=*)])≫			
	([CX3,c;R:1](=[OX1])[NX3;R]([C,c:5].[C,c:6]-*)[CX3,c;R:2](=[OX1]).[CX3,c;R:3](=[OX1])[NX3;R](-*)[CX3;R:4](=[OX1]))			
21	polyurethane	di/polyisocyanate	di/polyol	polyaddition
	([NX2:1]=[CX2]=[OX1,SX1:2].[NX2:3]=[CX2:4]=[OX1,SX1:5]).			
	([OX2,SX2;H1;!$([O,S]C=*):6].[OX2,SX2;H1;!$([O,S]C=*):7])≫			
	(*-[CX3](=[OX1,SX1:2])[NX3:1].[NX3:3][CX3:4](=[OX1,SX1:5])[OX2,SX2;!$([O,S]C=*):6].[OX2,SX2;!$([O,S]C=*):7]-*)			
22	polyoxazolidone	di/polyepoxide	di/polyisocyanate	polyaddition
	([CX4;H2,H1,H0;R:1]1[OX2;R:2][CX4;H1,H0;R:3]1.[CX4;H2,H1,H0;R:4]2[OX2;R:5][CX4;H1,H0;R:6]2).			
	([OX1,SX1:7]=[CX2:8]=[NX2:9].[OX1,SX1:10]=[CX2:11]=[NX2:12])≫			
	(*-[OX2:2][CX4:3]([CX4:1]-*).[CX4;R:6]1[OX2;R:5][CX2;R:8](=[OX1,SX1:7])[NX3;R:9][CX4;R:4]1.			
	[OX1,SX1:10]=[CX3:11](-*)[NX3:12](-*))			

a The 7 different polymer classes can be generated by the 22 polymerization rules.

b Bipolymer.

c A monomer with two different functional groups for the same polymerization reaction within the same molecular entity.

d Polycarbonate.

e Synthetic equivalent of phosgene.

f Oxidative carbonylation in the case of CO.

g Usually oxidative polymerization was applied.

h Polyethersulfone (PES) was generated.

i Polyetherketone (PEK) or polyetheretherketone was generated.

Chain polymerization is applied to a single starting monomer having at least one polymerizable functional group. Step-growth polymerization reactions are applied to two starting monomers with at least two polymerizable functional groups within each molecular entity or a single monomer with two different functional groups for the same polymerization reaction.

### Selection and Mapping Monomers to Polymerization Reactions

First, from the given set of starting molecules, we select those that can form monomers in the polymerization reaction rule set according to the presence or absence of the class-specific functional groups. The monomer or monomer pair is then mapped to an applicable polymerization reaction depending on the presence or absence of polymerizable functional groups in the reaction rule. The polymerizable functional groups in the rule set include vicinal and/or geminal functional groups as summarized in Table 1. Here, starting molecules containing incompatible functional groups are automatically excluded from the calculation.

In this study, 1083 starting molecules were manually extracted from the literature and lists of commercially available compounds. Their chemical structures were expressed in SMILES strings.^25^ SMiPoly’s submodule “monc.py” classified the 1083 compounds into 19 monomer classes according to the presence or absence of the class-specific functional groups. In the step-growth polymerization, the upper and lower limits for the occurrence number of a polymerizable functional group in a given monomer were limited to the default values of 2 and 4, respectively. The classification result is summarized in Tables 2 and 3, respectively.

**Table 2:** Result of Monomer Classification

monomer class	no. of compounds
vinyl	462
cyclic olefin	52
epoxid	71
lactone	13
lactam	1
hydroxy carboxylic acid	7
amino acid	1
hindered phenol	8
bis(*p-*halogenated aryl) sulfone	4
bis(*p-*fluoroaryl)ketone	1
di/poly epoxide	14
di/polycarboxylic acid	85
di/polyol (include thiol)	162
di/polyol (without thiol)	132
di/polyamine	149
primary di/polyamine	143
di/polyisocyanate	19
cyclic carboxylic acid ahnydride	33
di/polycyclic catboxylic acid anhydride	28

**Table 3:** Number of SMiPoly-Generated Polymers Classified into Seven Polymer Classes

polymer class	no. of generated polymers
polyolefin	523,080
polyester	25,204
polyether	868
polyamide	15,554
polyimide	5,445
polyurethane	4,200
polyoxazolidone	486
total	574,837

### Library Generation

To generate a virtual polymer library, an applicable set of polymerization reactions is performed according to the classification of a given monomer set. Polymerizable functional groups identified by “polg.py” are denoted by asterisks. If necessary, an option can be specified to limit the class of polymers generated. The generator lists all applicable monomer combinations for a given polymerization reaction according to the pre-computed monomer class and converts one or two starting monomers to products. The polymerization of polyolefins by addition chain polymerization and polyesters and polyamides composed of monomers with two different functional groups for the same polymerization reaction within the same molecular entity produces bipolymers as well as homopolymers. Polymers with regioisomeric structures were treated as different compounds. The relation between monomer classes, polymerization reactions, and polymer classes is illustrated in Figure S1.

## Results and Discussion

**Figure 3:**
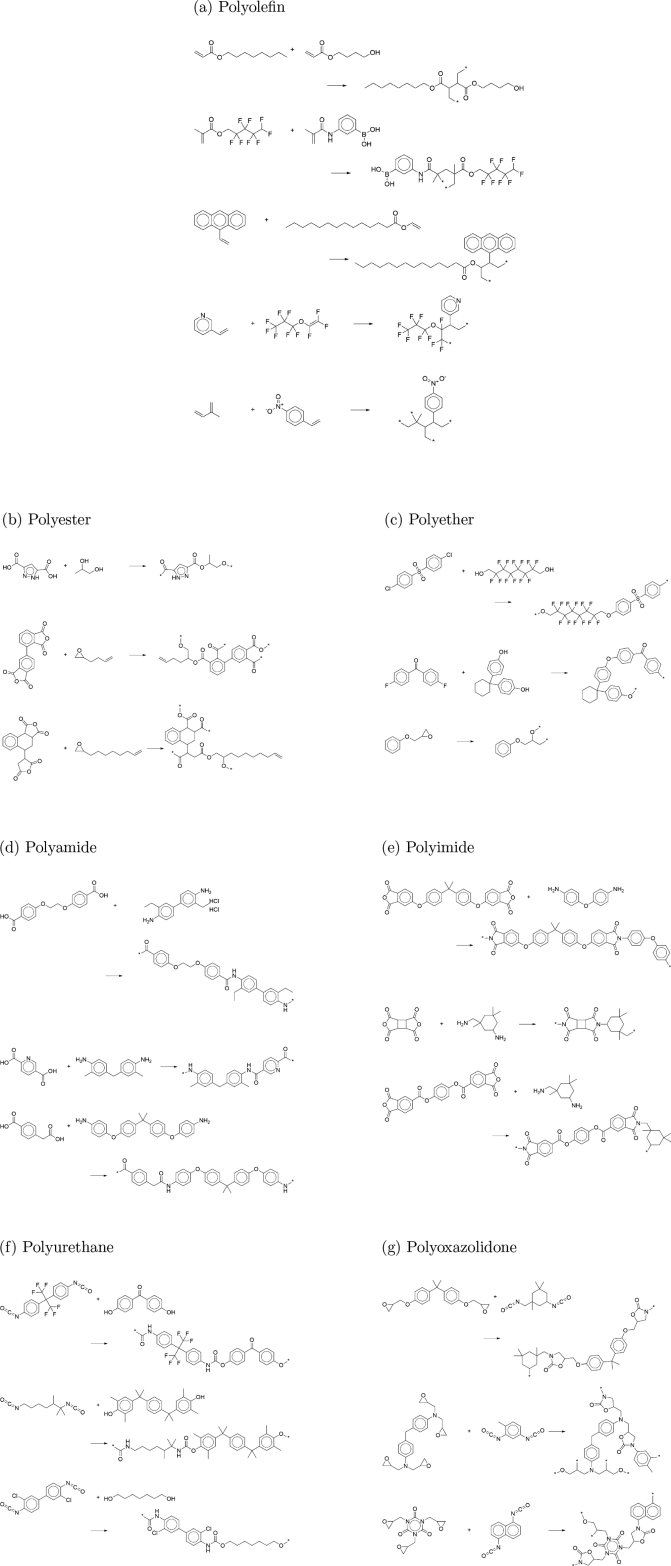
Examples of generated virtual polymers belonging to seven different polymer classes and their polymerization reactions: (a) polyolefin, (b) polyester, (c) polyether, (d) polyamide, (e) polyimide, (f) polyurethane, and (g) polyoxazolidone.

As summarized in Table 2, the 1083 starting molecules were classified into one or more of the 19 different monomer classes according to the presence or absence of the class-specific reaction sites. From these monomers, a total of 574,837 virtual polymers were generated by applying the 22 reaction rules. Here, polymers with the same repeating units but different starting monomers were distinguished. These virtual polymers constituted the seven polymer classes (polyolefin, polyester, polyether, polyamide, polyimide, polyurethane, and polyoxazolidone) as summarized in Table 3. Each of these polymers is defined by a single repeating unit because the current virtual library consists of either homopolymers or alternation copolymers. The class of polyolefins is given by homopolymers and binary alternating copolymers that are generated from all combinations of the olefin and/or cyclic olefin monomers. The classes of polyesters and polyamides consist of homopolymers and binary alternating copolymers generated from the combinations of hydroxy or amino carboxylic acid. The other polymer classes are all composed of homopolymers. Figure 3 depicts several examples of generated polymers for each polymer class along with their polymerization reactions. The Supporting Information provides more randomly selected examples that belong to the seven different polymer classes (Figure S2).

**Figure 4:**
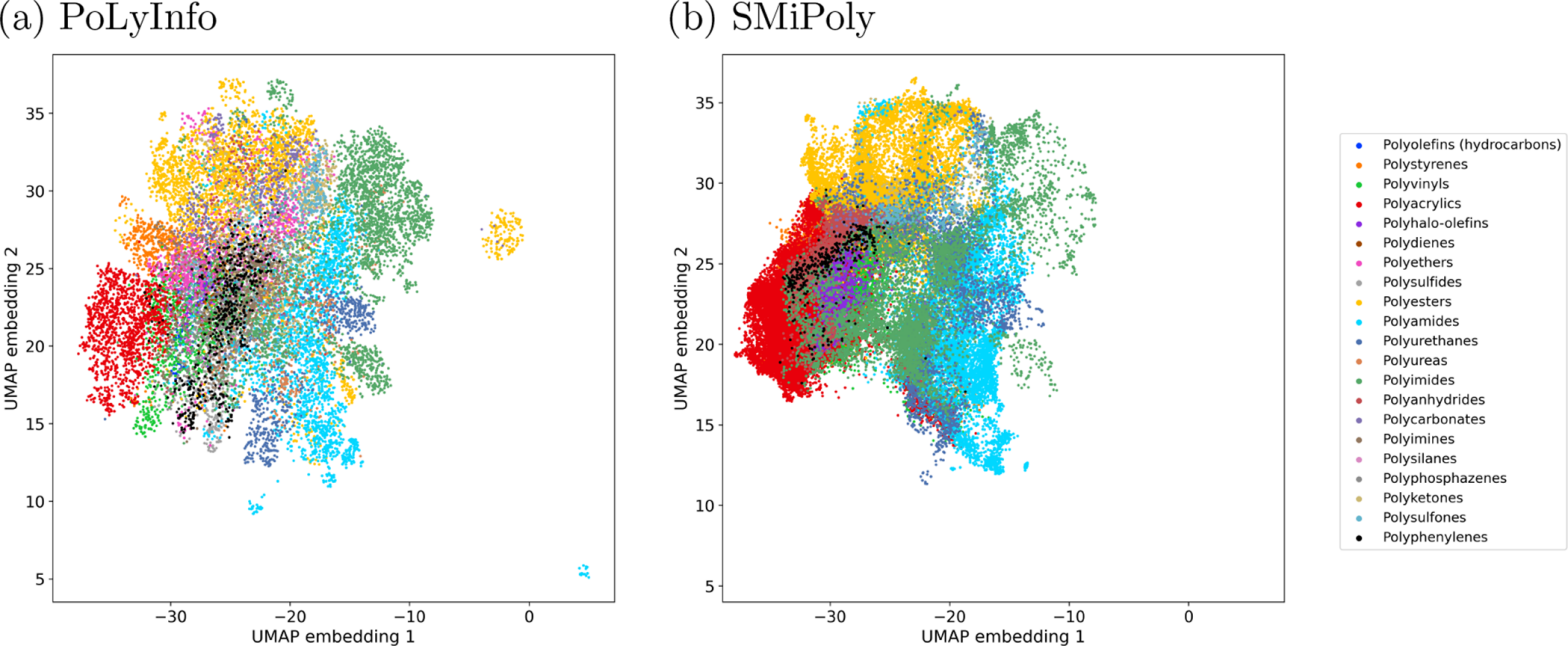
UMAP projection of all polymers with their chemical structures in (a) PoLyInfo (16,223 polymers) and (b) SMiPoly virtual library (169,347 polymers). ECFP6 descriptor, which was constructed after generating the macrocyclic oligomer with 10-mer of the repeating unit, was used as input of the UMAP. The 21 classes of the polymer backbones are color-coded according to the classification of RadonPy.

We investigated the degree of overlap or coverage and novelty between the distributions of polymers synthesized to date and the virtually created polymers. As the set of existing polymers, 16,223 polymers were extracted from PoLyInfo, the world’s largest database of synthetic polymers. The uniform manifold approximation and projection (UMAP)^47^ of all polymers with their chemical structures in PoLyInfo and the SMiPoly virtual library is displayed in Figure 4, which shows that SMiPoly is generally able to cover the distribution of existing polymers. A more detailed, quantitative evaluation was performed for each of the seven polymer classes as described below.

PoLyInfo defines 21 different polymer classes based on its own classification criteria. The polymer classes are identified by the first three letters of the polymer ID (referred to as PID in PoLyInfo), such as P01, P02, P03, etc. Here, the definitions of polymer classes in SMiPoly and PoLyInfo do not exactly match. To eliminate ambiguity in the classification criteria, the polymers generated by SMiPoly and those recorded in PoLyInfo were reclassified to 21 classes using the classifier function (poly.polyinfo_classifier) in the Python library RadonPy,^48^ which mimics the classification criterion of PoLyInfo’s 21 classes. The six classes of RadonPy that were consistent with SMiPoly and PoLyInfo were used for comparison. The correspondence of the six classes with the RadonPy’s 21 classes, SMiPoly’s seven classes, and PoLyInfo’s 21 classes is summarized in Table 4. In addition, only polymers with two asterisks in the SMILES string of the repeating unit, i.e., linear homopolymers and alternating copolymers, were considered for comparison because it is difficult to represent the branching and cross-linking polymers with SMILES. Further elimination of structural redundancy reduced the number of polymers produced by SMiPoly to 169,347 using the “poly.full_match_smiles_listself” function of RadonPy.^48^

**Table 4:** Correspondence of Polymer Classes between Existing Polymers in PoLyInfo, 21 Classes of RadonPy, and Seven Classes of SMiPoly-Generated Polymers

polymer class	RadonPy	PoLyInfo PID^a^	SMiPoly	no. of polymers in PoLyInfo	no. of polymers in SMiPoly
polyolefin	hydrocarbon of polyolefin	P01, P31	polyolefin	3,139	139,990
	polystyrene	P02, P32			
	polyvinyl	P03, P33			
	polyacrylate	P04, P34			
	halogenated polyolefin	P05, P35			
	polydiene	P06, P36			
polyester	polyester	P09, P39	polyester	2,775	9,903
polyether	polyether	P07, P37	polyether	4,692	5,547
polyamide	polyamide	P10, P40	polyamide	5,059	14,402
polyimide	polyimide	P13, P43	polyimide	2,755	6,553
polyurethane	polyurethane	P11, P41	polyurethane	695	2,772
			polyoxazolidone		
no. of unique polymers				16,223	169,347

a The first three letters of PID in PoLyInfo.

**Figure 5:**
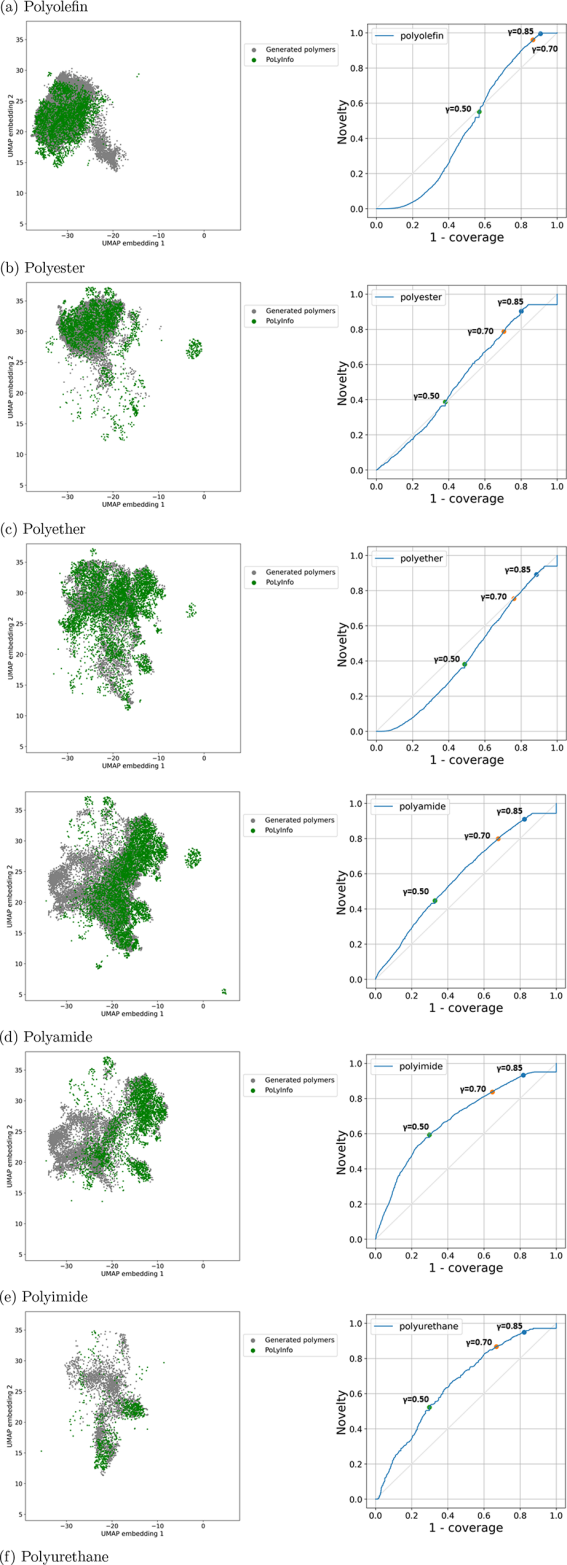
For each class of polymers, the coverage and novelty of the virtual library relative to existing polymers are evaluated based on CN curves and visual inspection of UMAP projections of the two sets of chemical structures: (a) polyolefin, (b) polyester, (c) polyether, (d) polyamide, (e) polyimide, and (f) polyurethane.

**Figure 6:**
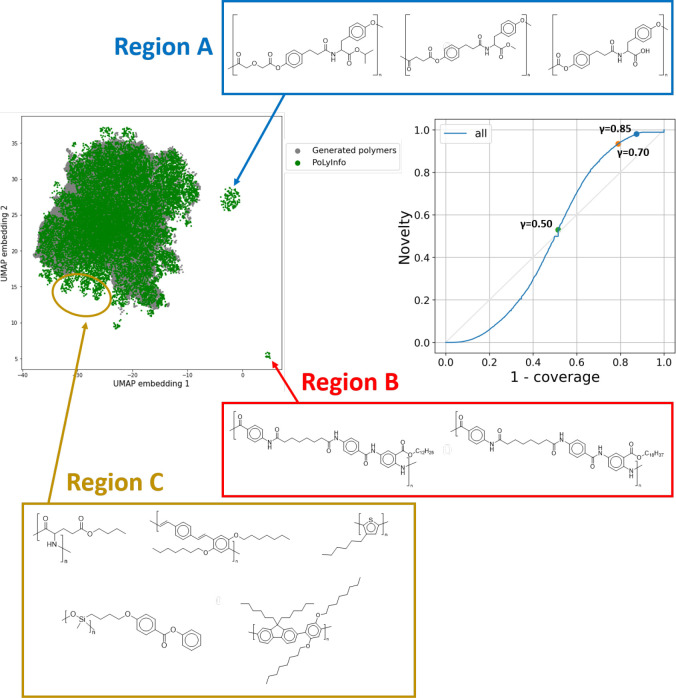
Coverage and novelty of virtual libraries with respect to existing polymers are evaluated based on the CN curve and visual inspections of the distribution of polymers’ chemical structures constituting the two sets by UMAP projections.

Here, we denote the sets of actually synthesized and virtual polymers belonging to a certain polymer class by  and , respectively. We assessed the coverage and novelty of  with respect to  as follows:

(1) Evaluate the pairwise similarity between all polymers in  and . As the similarity measure, we employed the Tanimoto coefficient, in which the chemical structure of each polymer was transformed into a 2048-bit vector with an extended connectivity fingerprint with a radius of three atoms (ECFP6).^49^ To consider the repeating structure of polymers, the ECFP6 descriptor was constructed after generating the macrocyclic oligomer with 10-mer of the repeating unit using RadonPy.^48^
(2) Set the threshold values of the Tanimoto coefficient as γ ∈ {0, 0.1, ..., 0.9, 1}.
(3) Coverage: calculate the percentage of polymers in  with a similarity greater than γ to those in .
(4) Novelty: calculate the percentage of polymers in  with a similarity less than γ to those in .
(5) Vary the threshold γ from 0 to 1, and draw a curve representing the balance between (1-coverage) and novelty (coverage–novelty (CN) curve) as shown in Figures 5 and 6.

The CN curve shows an upward or downward convex pattern depending on the inclusive relationship of the distributions of  and . If the two distributions are nearly coincident, the CN curve is drawn on the 45° line. If the distribution of  encompasses , the CN curve deviates slightly from the 45° line and shows an upward convex pattern. This is the most desirable virtual library, given that it contains reasonably novel and diverse polymers while maintaining a high coverage of existing ones. Conversely, if the virtual library set fails to reproduce a part of the synthetic polymers, the CN curve shows downward convexity. This is visually illustrated in Figure S3.

Figure 5 summarizes the CN curves for each of the six polymer classes and the distributions of  and  visualized on a two-dimensional plane by performing UMAP.^47^ The virtual libraries for polyamide, polyimide, and polyurethane, which exhibited moderately upward convex CN curves, achieved well-balanced coverage and novelty. When the threshold of Tanimoto similarity was set to γ ≥ 0.5, the coverage and novelty were approximately 67 and 45% for polyamide, 70 and 59% for polyimide, and 70 and 52% for polyurethane, respectively. The CN curves for polyether showed a slightly downward convex pattern. The coverage and novelty were 51 and 38% when the threshold was set to γ ≥ 0.5.

We also investigated the coverage with respect to all 16,223 polymers in PoLyInfo (Figure 6). The coverage and novelty of the 169,347 virtual polymers for all these polymers were 48 and 53% under γ ≥ 0.5, respectively.

The performance of the rule-based virtual polymer generator depends on the diversity of the starting monomer set and the comprehensiveness of polymerization reaction rules. As shown by the UMAP projection in Figure 6, the current SMIPoly virtual library clearly failed to cover some regions of the existing polymer distribution in PoLyInfo, which are denoted by regions A–C (see example polymers belonging to each region in Figure S4 in the Supporting Information). The presence of region A was due to the fact that the current SMiPoly does not implement the condensation reaction of hydroxycarboxylic acids and amino acids even though both types of monomers were included when the library was created. Region B arose because the starting monomer set did not include the relevant molecules; as shown in Table 2, the starting monomer set contains very few amino acid molecules. The polymers belonging to region C were mainly polyarylenes. The current SMiPoly could not cover this area because polymerization reactions with aryl coupling reactions and their monomers were undefined. This is the main reason for the low coverage and novelty in the generation of the class of polyethers. The class of polyethers defined by PoLyInfo (P07 and P37) includes polyoxides with chain structures as well as polymers with cyclic structures including C–O–C bonds and acetal structures in the main chain, but the current SMiPoly does not support these polymerization reactions or monomers.

## Conclusions

To generate synthesizable virtual polymer candidates, we have developed the Python library SMiPoly that implements 22 chemical rules for polymerization reactions. For any given set of starting molecules, the generator creates synthetic products by conducting automatically selected polymerization reactions applicable to the input monomers. All polymers produced are potentially synthesizable.

In this study, using 1083 available monomers, we generated 169,347 unique linear polymers forming seven different types of polymers, such as polyolefin, polyester, polyether, polyamide, polyimide, polyurethane, and polyoxazolidone. The reaction space that can be represented by the current release of SMiPoly covers approximately 50% (γ ≥ 0.5) of the entire chemical space of polymers synthesized to date. When focusing on nitrogen containing polymers defined by SMIPoly, it covered 70% of the corresponding chemical space with around 50% novelty (γ ≥ 0.5). The coverage can be monotonically increased by further expanding the list of raw monomers and the set of polymerization reactions.

Here, we remark on a comparison with the OMG virtual polymer database, which was created using a rule-based polymerization algorithm similar to SMiPoly; OMG contains approximately 12 million virtual polymers generated from 17 different polymerization reaction rules using approximately 80,000 small molecules as starting materials. Table S2 in the Supporting Information summarizes whether each of the 21 polymer classes of PoLyInfo can be generated by SMiPoly and OMG. Currently, SMiPoly implements 59% (10/17) of the reaction rules defined in the OMG, and the OMG implements 36% (8/22) of the reaction rules defined in SMiPoly.

Finally, the limitations of the current version of SMiPoly are summarized. For example, it is necessary to extend the rule set of polymerization reactions, such as ring-opening metathesis polymerization (ROMP), addition-condensation polymerization to generate phenol and/or melamine resin, and main-chain growth with aryl coupling reaction. The difficulty in defining these polymerization reactions arises from the complexity of defining the monomer structures to be reacted. In addition, in order to improve the accuracy of monomer selection and polymerization reaction prediction, olefin monomers need to be further classified in detail. More importantly, although branched polymers play an important role in the development of thermosetting polymers, the generation of branched polymers cannot be handled by the current version of SMiPoly. Since this limitation is due to SMILES-based modeling, it is necessary to introduce a structural representation that can handle stochastic polymer structures such as BigSMILES.^50^

## Data Availability

The source code of SMiPoly is available from the GitHub website(https://github.com/PEJpOhno/SMiPoly). A sample script to generate the polymer library is in the directory named “sample_script”. The list of starting molecules is stored in the directory “sample_data”. The list of the selected monomers and generated polymers is also available.

## Supporting Information Available

Additional experimental details, materials, and methods, including (1) functional group transformations in the polymerization reaction rules (Table S1), (2) monomer class, polymerization reaction, and polymer class (Figure S1), (3) generated polymers belonging to seven different polymer classes and their polymerization reactions (Figure S2), (4) drawing CN curve (Figure S3), (5) examples of polymers belonging to Regions A–C in Figure 6 (Figure S4), and (6) comparison of polymerization reaction rule sets between SMiPoly and OMG (Table S2) (PDF)
