# Supplementary material for: SMiPoly: Generation of a Synthesizable Polymer Virtual Library Using Rule-Based Polymerization Reactions
Source: J Chem Inf Model. 2023 Aug 21;63(17):5539–48. doi: 10.1021/acs.jcim.3c00329 (PMC10498440; doi:10.1021/acs.jcim.3c00329)
Supplement: Supplementary file 1 — ci3c00329_si_001.pdf [file ci3c00329_si_001.pdf]

Supporting Information: SMiPoly: Generation of Synthesizable Polymer Virtual Library using Rule-based Polymerization Reactions

Mitsuru Ohno,[*,†] Yoshihiro Hayashi,[‡,¶] Zhang Qi,[‡] Yu Kaneko,[†] and Ryo Yoshida[*,‡,¶,§]

†*Daicel Corporation, Kita-ku, Osaka 530-0011, Japan*

‡*The Institute of Statistical Mathematics, Research Organization of Information and Systems, Tachikawa, Tokyo 190-8562, Japan*

¶*The Graduate University for Advanced Studies, SOKENDAI, Tachikawa, Tokyo 190-8562, Japan*

§*National Institute for Materials Science, Ibaraki, 305-0047 Japan*

E-mail: mt_ohno@jp.daicel.com; yoshidar@ism.ac.jp

Contents

1 **Functional group transformations in the polymerization reaction rules** **S-3**

2 **Monomer class, polymerization reaction, and polymer class** **S-5**

3 **Generated polymers belonging to seven different polymer classes and their polymerization reactions** **S-7**

4 **Drawing coverage–novelty curve** **S-21**

5 **Examples of polymers belonging to Regions A, B, and C in Figure 6** **S-22**

6 **Comparison of polymerization reaction rule sets between SMiPoly and Open Macromolecular Genome** **S-29**

References **S-31**

1 Functional group transformations in the polymerization reaction rules

Table S1: Illustrative examples of functional group transformations in the 22 polymerization reaction rules.

	polymer class	reaction formula
1	polyolefin	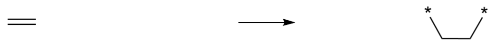
2	polyolefin	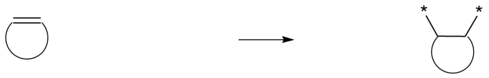
3	polyolefin	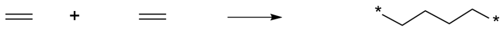
4	polyolefin	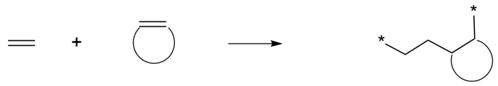
5	polyolefin	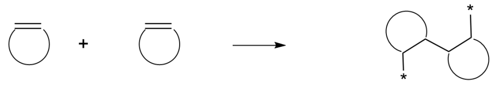
6	polyester	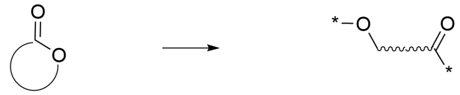
7	polyester	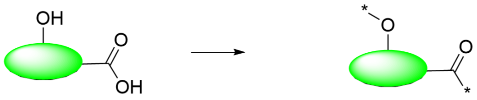
8	polyester	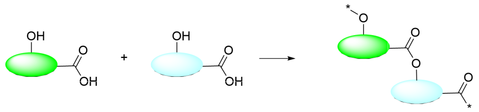
9	polyester	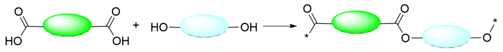
10	polyester	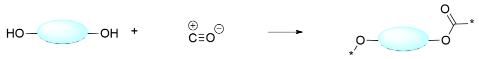
11	polyester	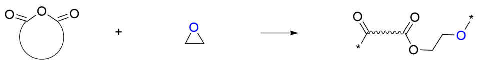
12	polyether	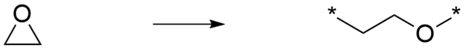
13	polyether	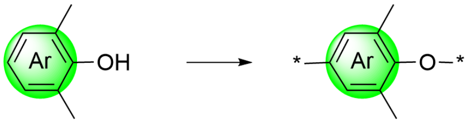
14	polyether	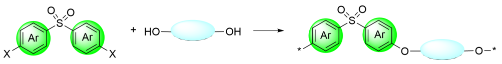
15	polyether	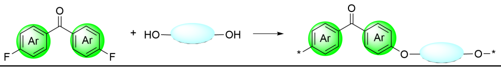

(Table S1: continued)

	polymer class	reaction formula
16	polyamide	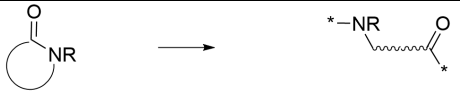
17	polyamide	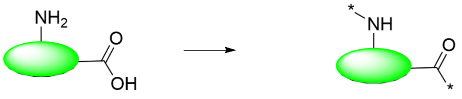
18	polyamide	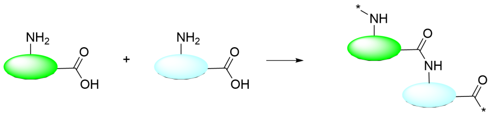
19	polyamide	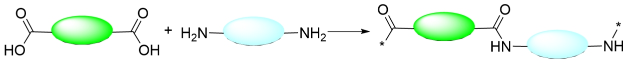
20	polyimide	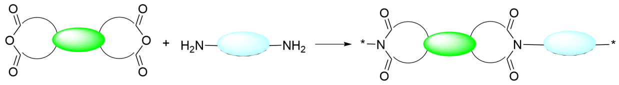
21	polyurethane	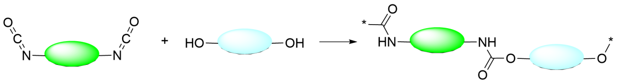
22	polyoxazolidone	22 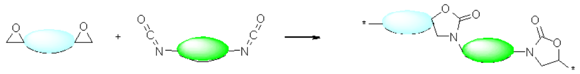

2 Monomer class, polymerization reaction, and polymer class

Figure S1 illustrates the relation between the monomer class, polymerization reaction, and the polymer class in the generation of polyesters. Polymerization reactions to synthesize polyesters include ring-opening chain polymerization, dehydration condensation, and self-condensation. The submodule “monc.py” classifies monomers extracted from inputted starting molecules into monomer classes according to the predefined rules. For the polyester formation, the monomer class of lactones, hydroxy carboxylic acids, the pair of diols and dicarboxylic acids, and the pair of epoxides and cyclic anhydrides are applicable. The submodule “polg.py” performs *in silico* polymerization to generate polyesters by applying the functional group transformations to the classified starting monomers. Here, the epoxide monomer class is used not only for the synthesis of polyesters but also for the synthesis of polyethers via the ring-opening chain polymerization.

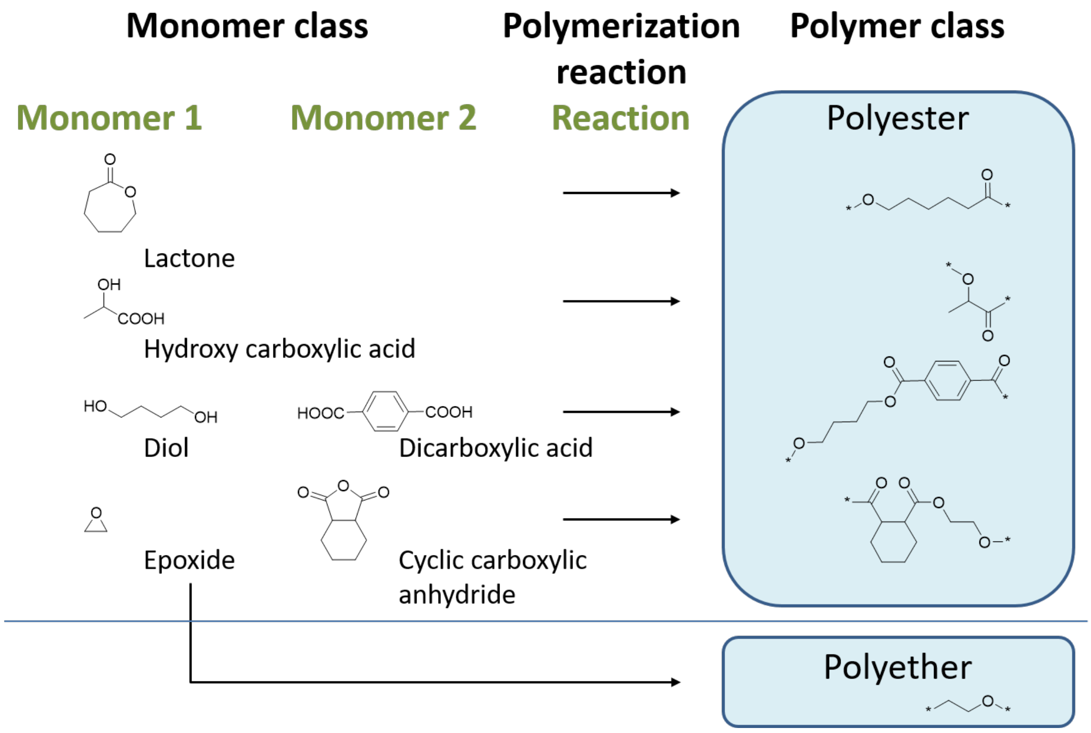

Figure S1: Illustration of monomer class, polymerization reaction, and polymer class to generate polyesters and polyether

3 Generated polymers belonging to seven different polymer classes and their polymerization reactions

(a) Polyolefin (1/2)

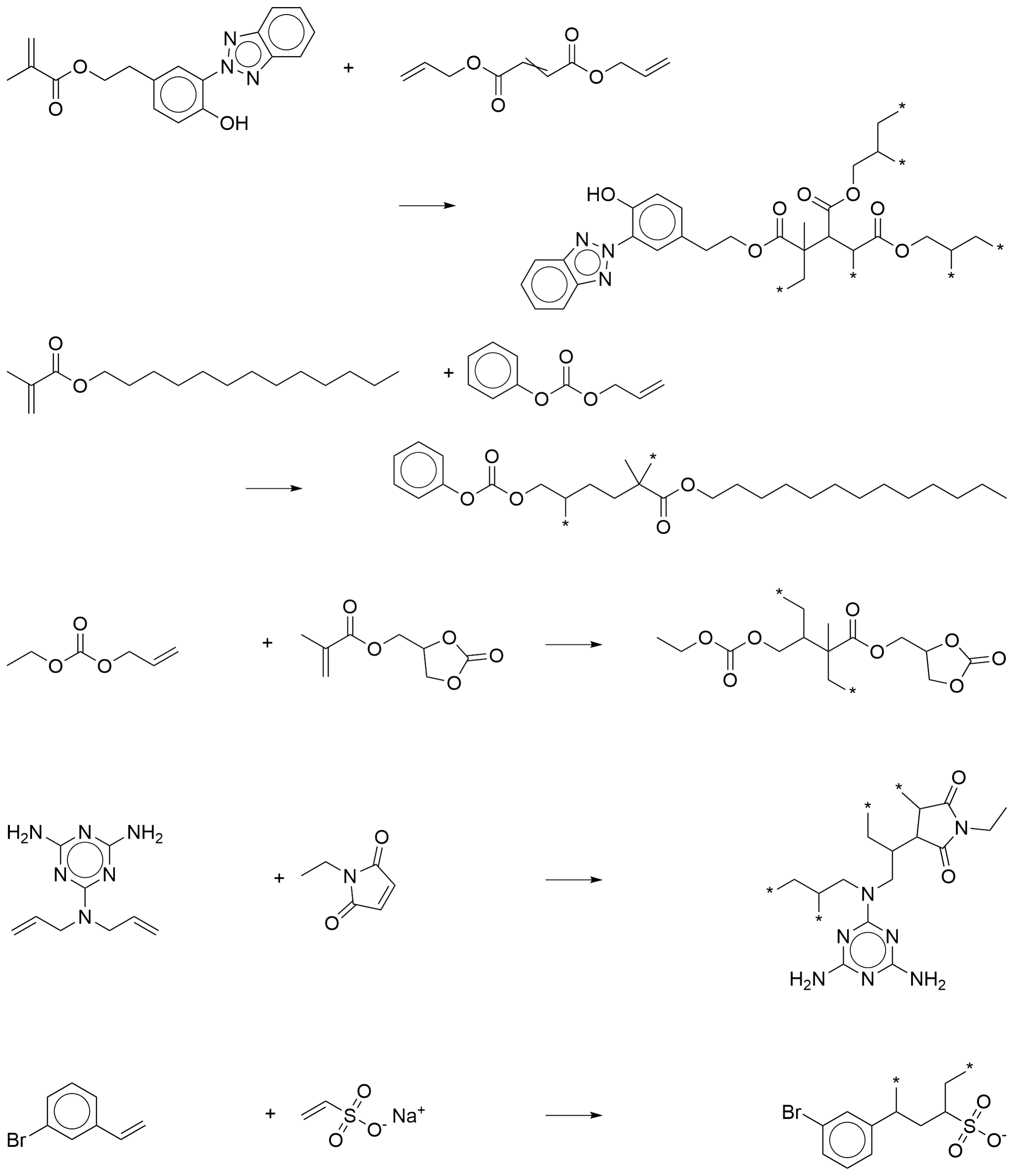

Figure S2: Examples of generated polymers belonging to seven different polymer classes and their polymerization reactions: (a) polyolefin, (b) polyester, (c) polyether, (d) polyamide, (f) polyimide and (g) polyoxazolidone *(continued)*.

(a) Polyolefin (2/2)

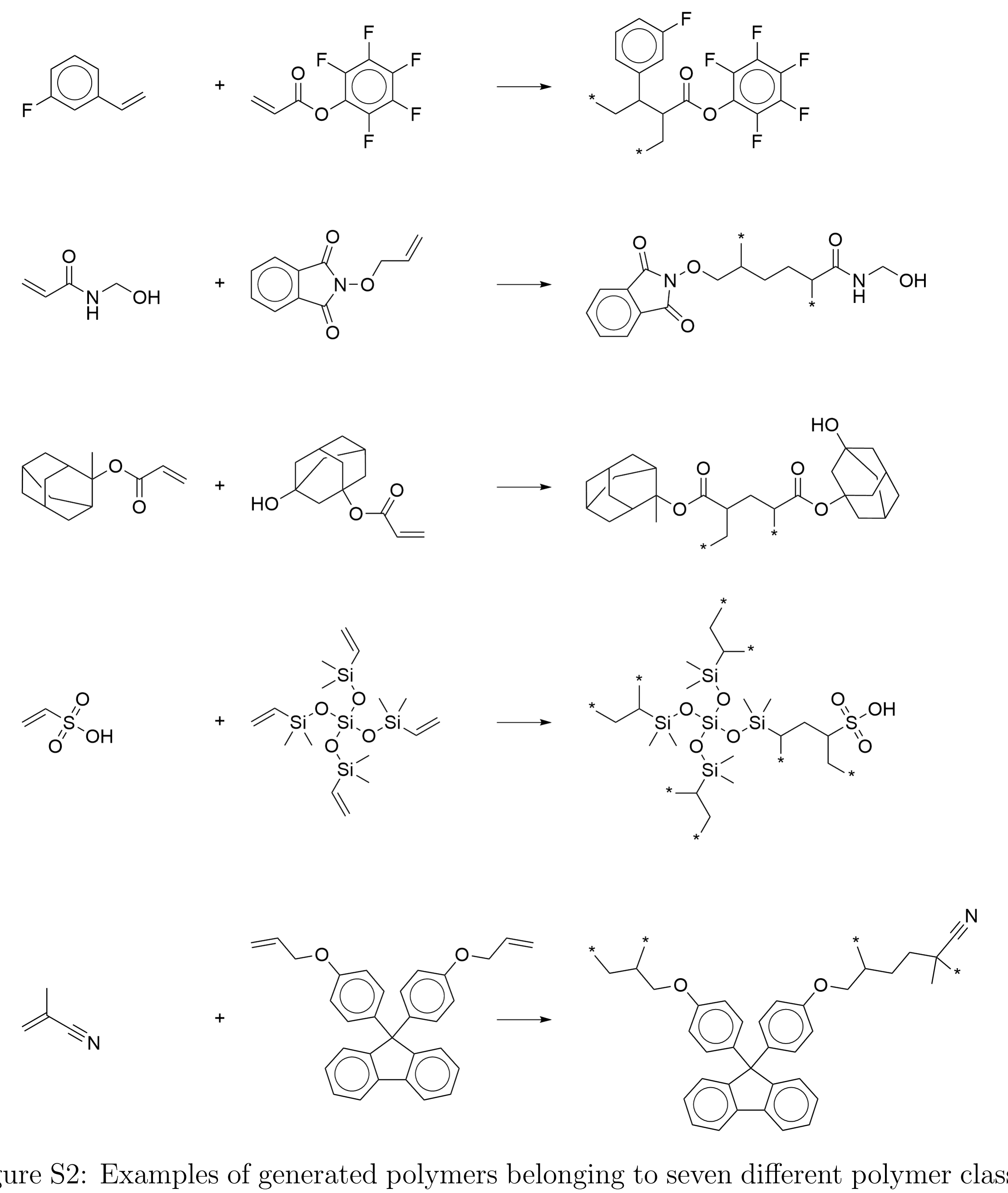

Figure S2: Examples of generated polymers belonging to seven different polymer classes and their polymerization reactions: (a) polyolefin, (b) polyester, (c) polyether, (d) polyamide, (f) polyimide and (g) polyoxazolidone *(continued)*.

(b) Polyester (1/2)

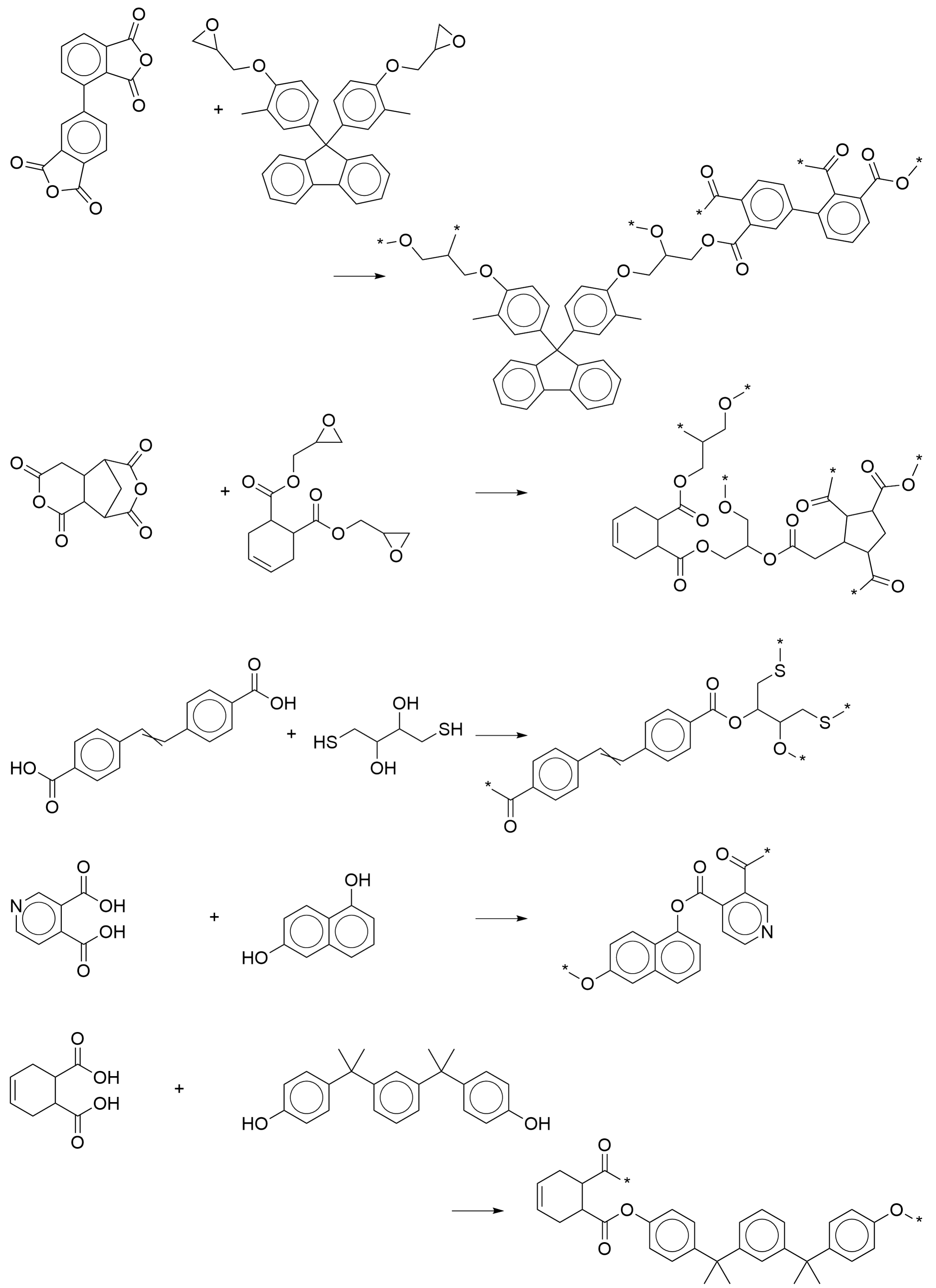

Figure S2: Examples of generated polymers belonging to seven different polymer classes and their polymerization reactions: (a) polyolefin, (b) polyester, (c) polyether, (d) polyamide, (f) polyimide and (g) polyoxazolidone *(continued)*.

(b) Polyester (2/2)

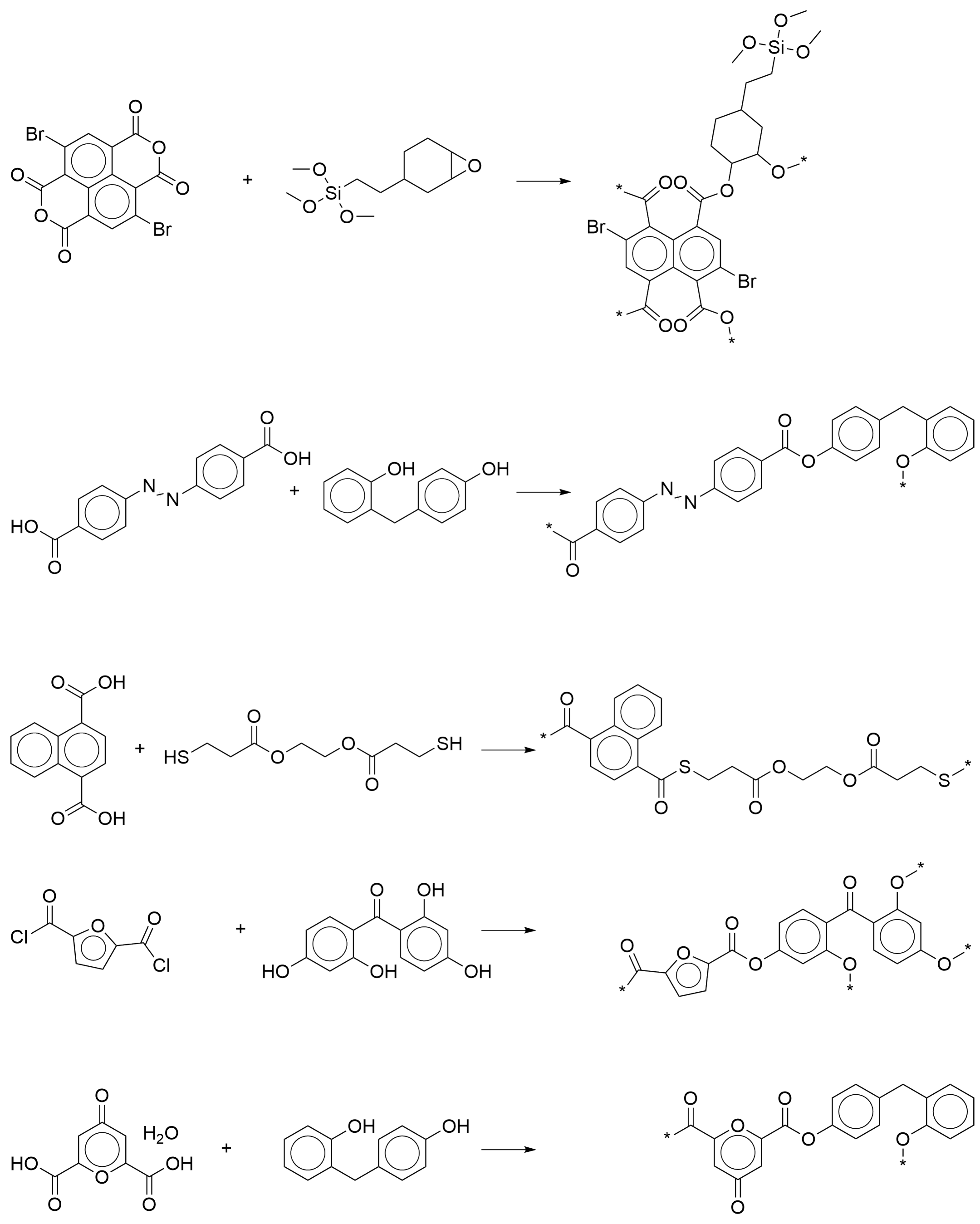

Figure S2: Examples of generated polymers belonging to seven different polymer classes and their polymerization reactions: (a) polyolefin, (b) polyester, (c) polyether, (d) polyamide, (f) polyimide and (g) polyoxazolidone *(continued)*.

(c) Polyether (1/2)

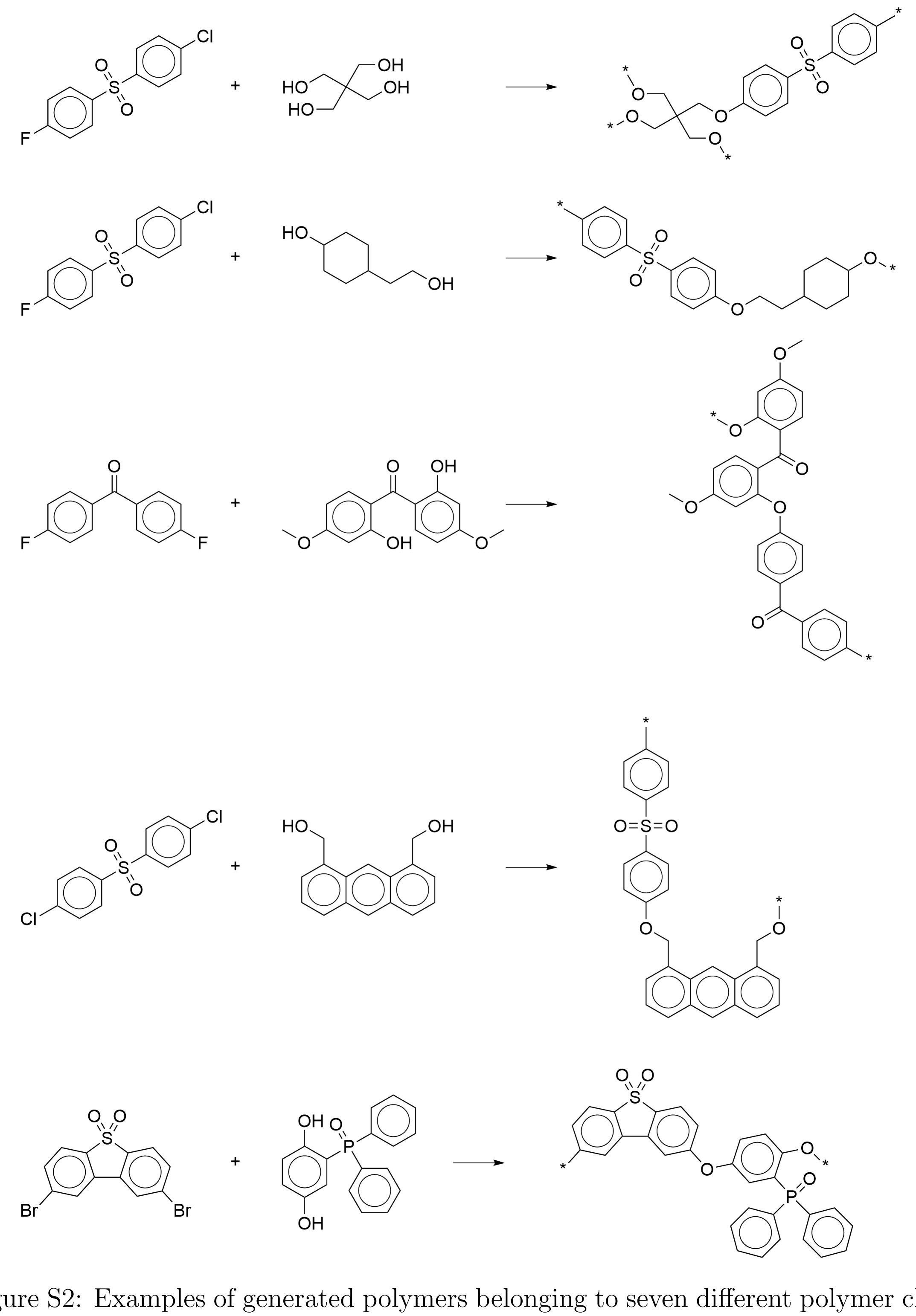

Figure S2: Examples of generated polymers belonging to seven different polymer classes and their polymerization reactions: (a) polyolefin, (b) polyester, (c) polyether, (d) polyamide, (f) polyimide and (g) polyoxazolidone *(continued)*.

(c) Polyether (2/2)

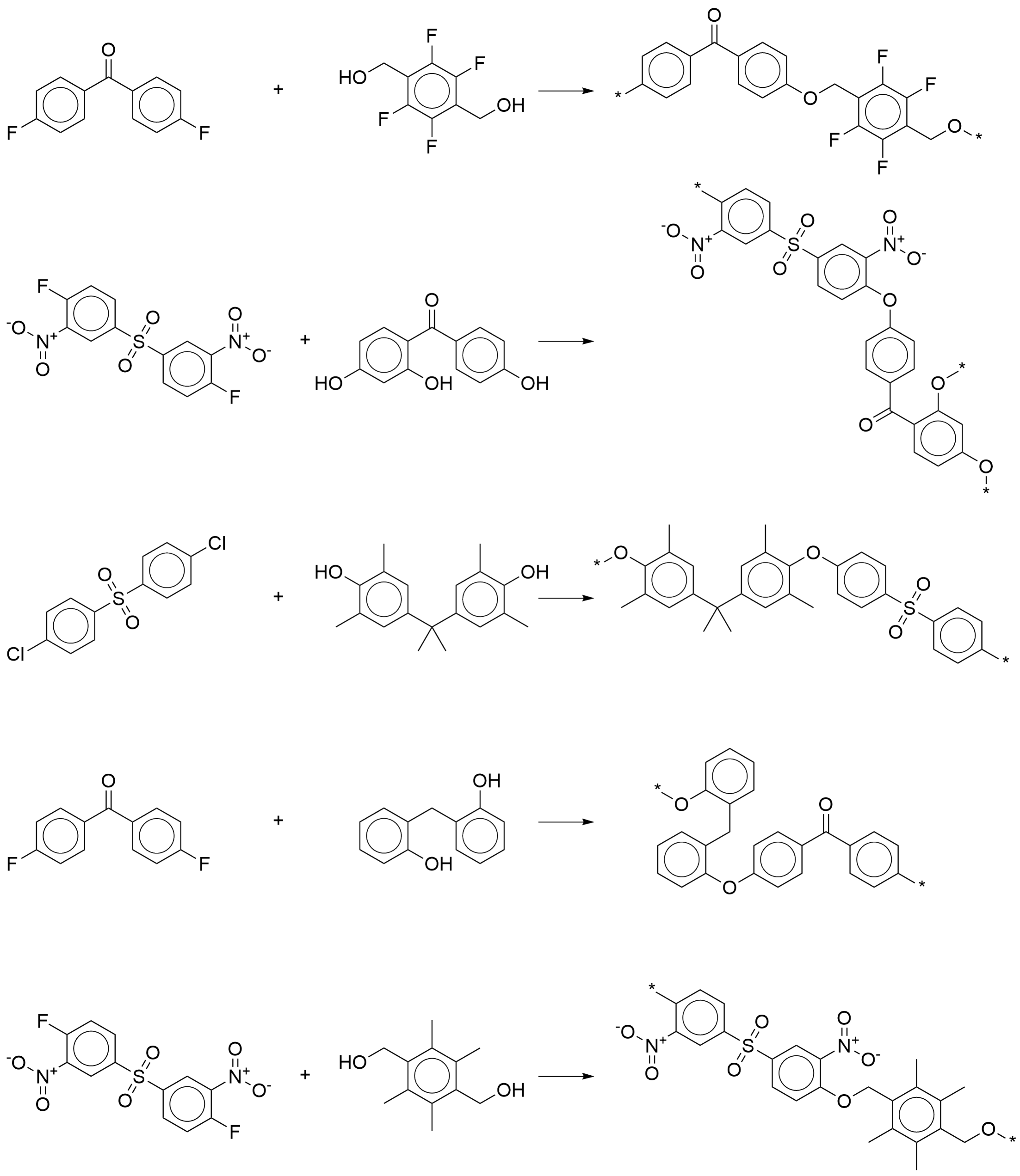

Figure S2: Examples of generated polymers belonging to seven different polymer classes and their polymerization reactions: (a) polyolefin, (b) polyester, (c) polyether, (d) polyamide, (f) polyimide and (g) polyoxazolidone *(continued)*.

(d) Polyamide (1/2)

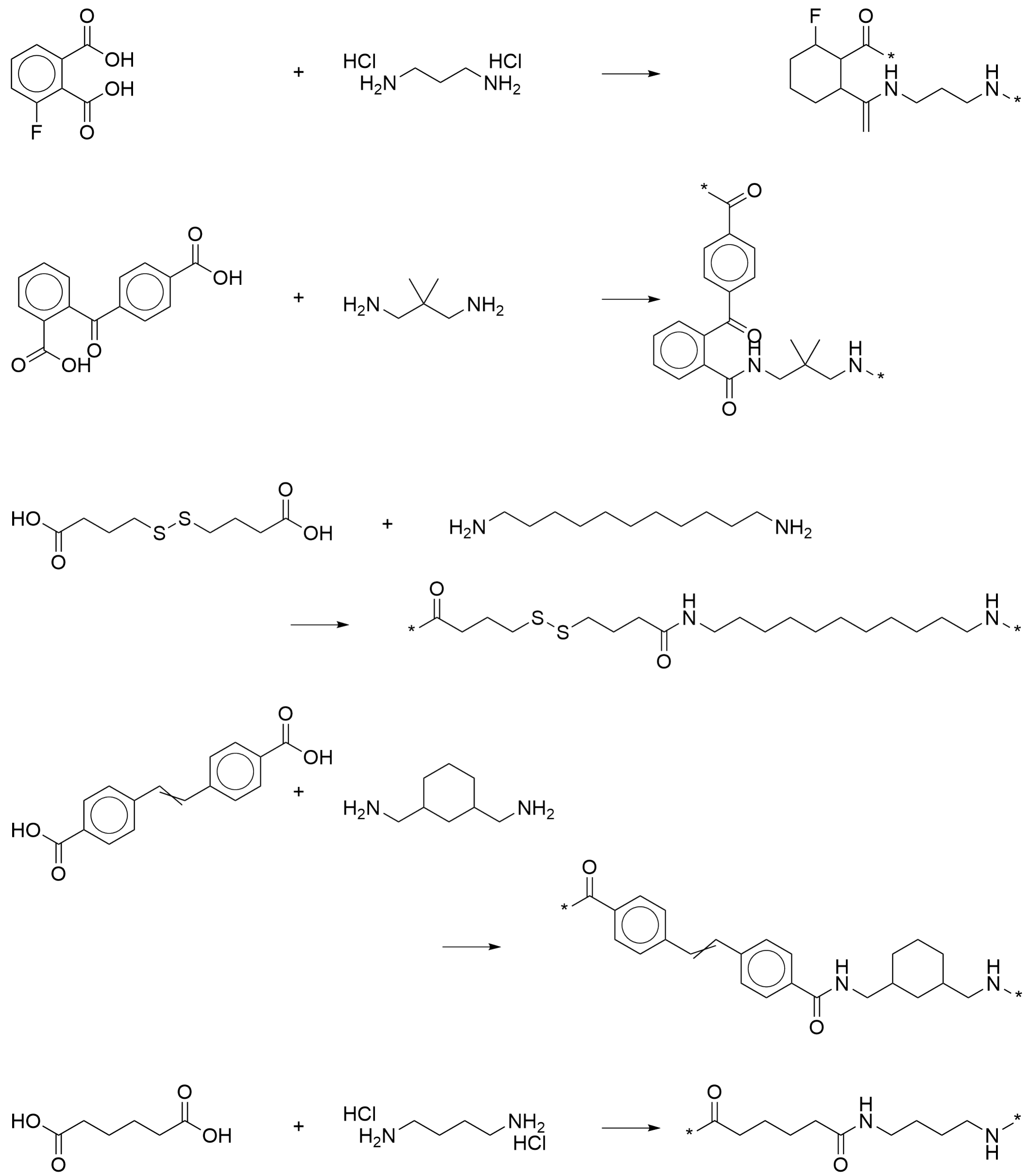

Figure S2: Examples of generated polymers belonging to seven different polymer classes and their polymerization reactions: (a) polyolefin, (b) polyester, (c) polyether, (d) polyamide, (f) polyimide and (g) polyoxazolidone *(continued)*.

(d) Polyamide (2/2)

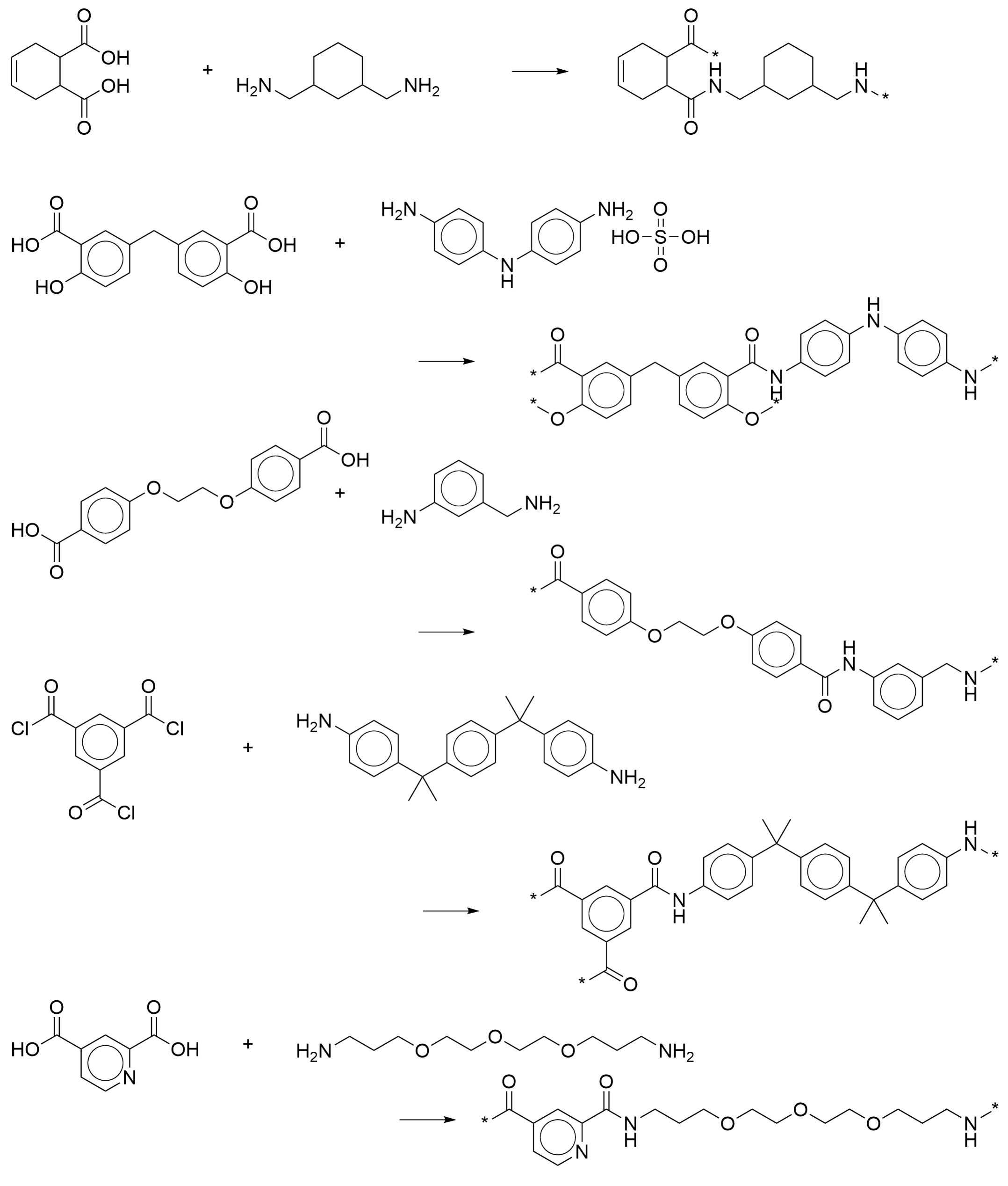

Figure S2: Examples of generated polymers belonging to seven different polymer classes and their polymerization reactions: (a) polyolefin, (b) polyester, (c) polyether, (d) polyamide, (f) polyimide and (g) polyoxazolidone *(continued)*.

(e) Polyimide (1/2)

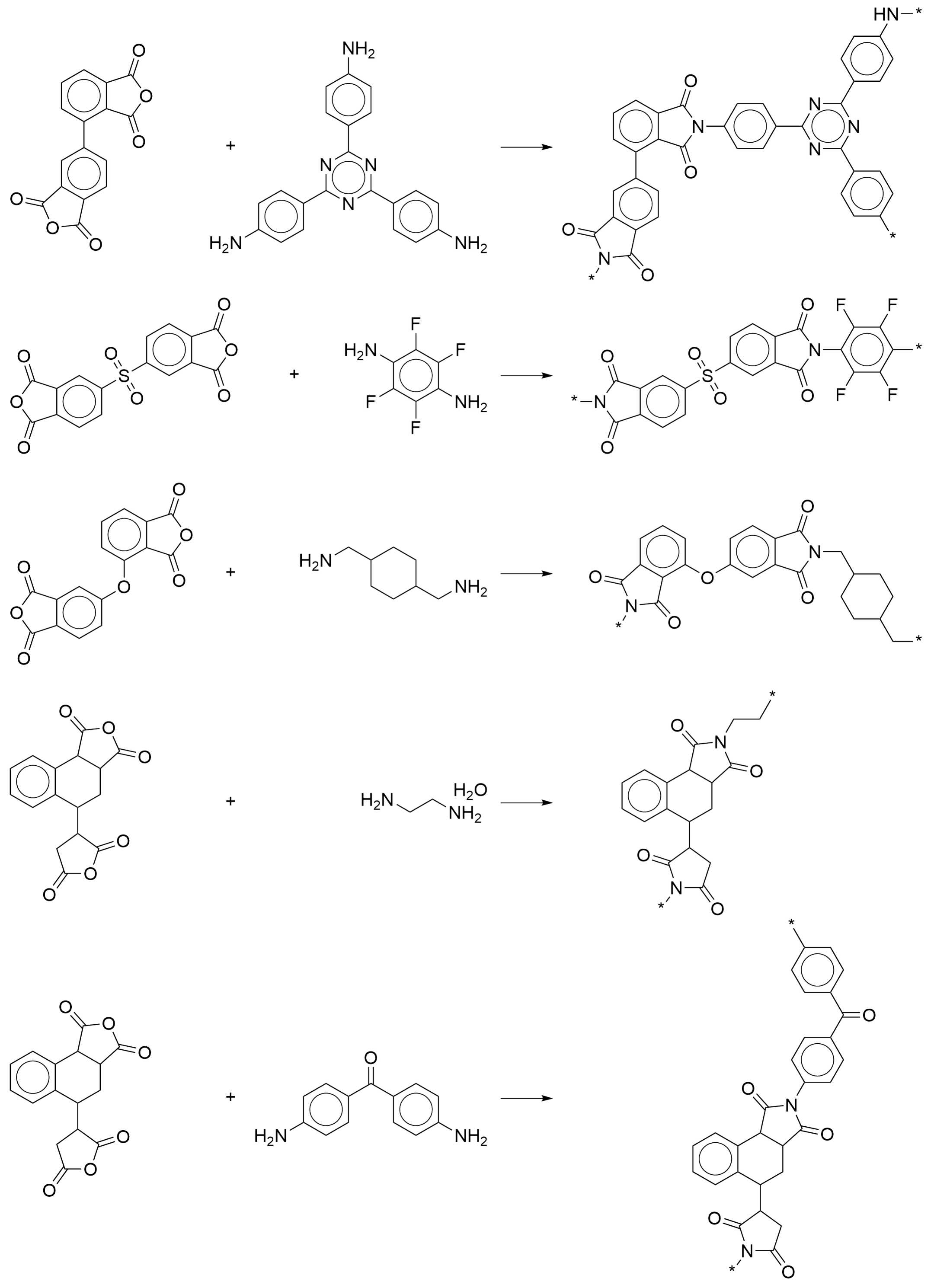

Figure S2: Examples of generated polymers belonging to seven different polymer classes and their polymerization reactions: (a) polyolefin, (b) polyester, (c) polyether, (d) polyamide, (f) polyimide and (g) polyoxazolidone *(continued)*.

(e) Polyimide (2/2)

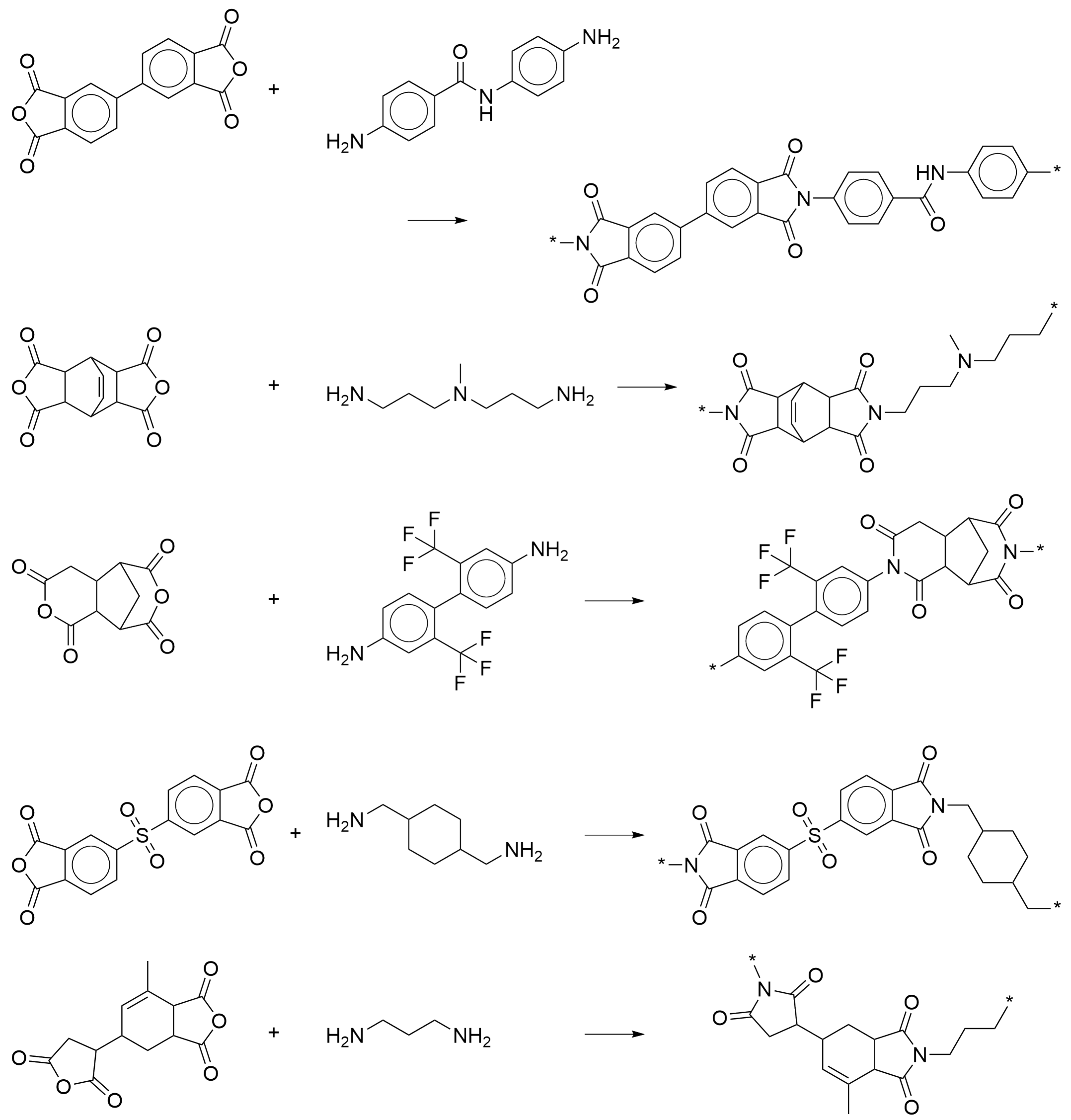

Figure S2: Examples of generated polymers belonging to seven different polymer classes and their polymerization reactions: (a) polyolefin, (b) polyester, (c) polyether, (d) polyamide, (f) polyimide and (g) polyoxazolidone *(continued)*.

(f) Polyurethane (1/2)

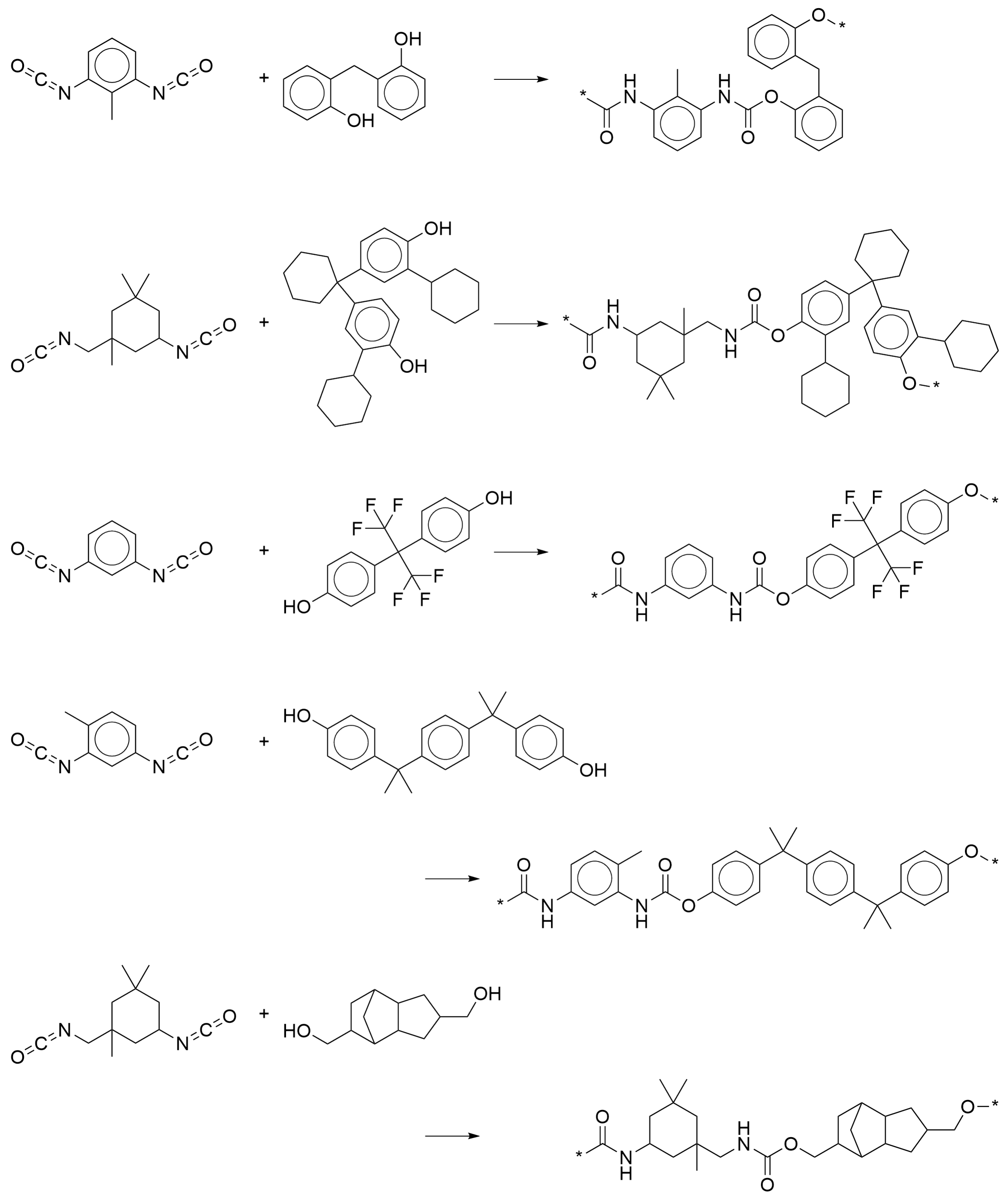

Figure S2: Examples of generated polymers belonging to seven different polymer classes and their polymerization reactions: (a) polyolefin, (b) polyester, (c) polyether, (d) polyamide, (f) polyimide and (g) polyoxazolidone *(continued)*.

(f) Polyurethane (2/2)

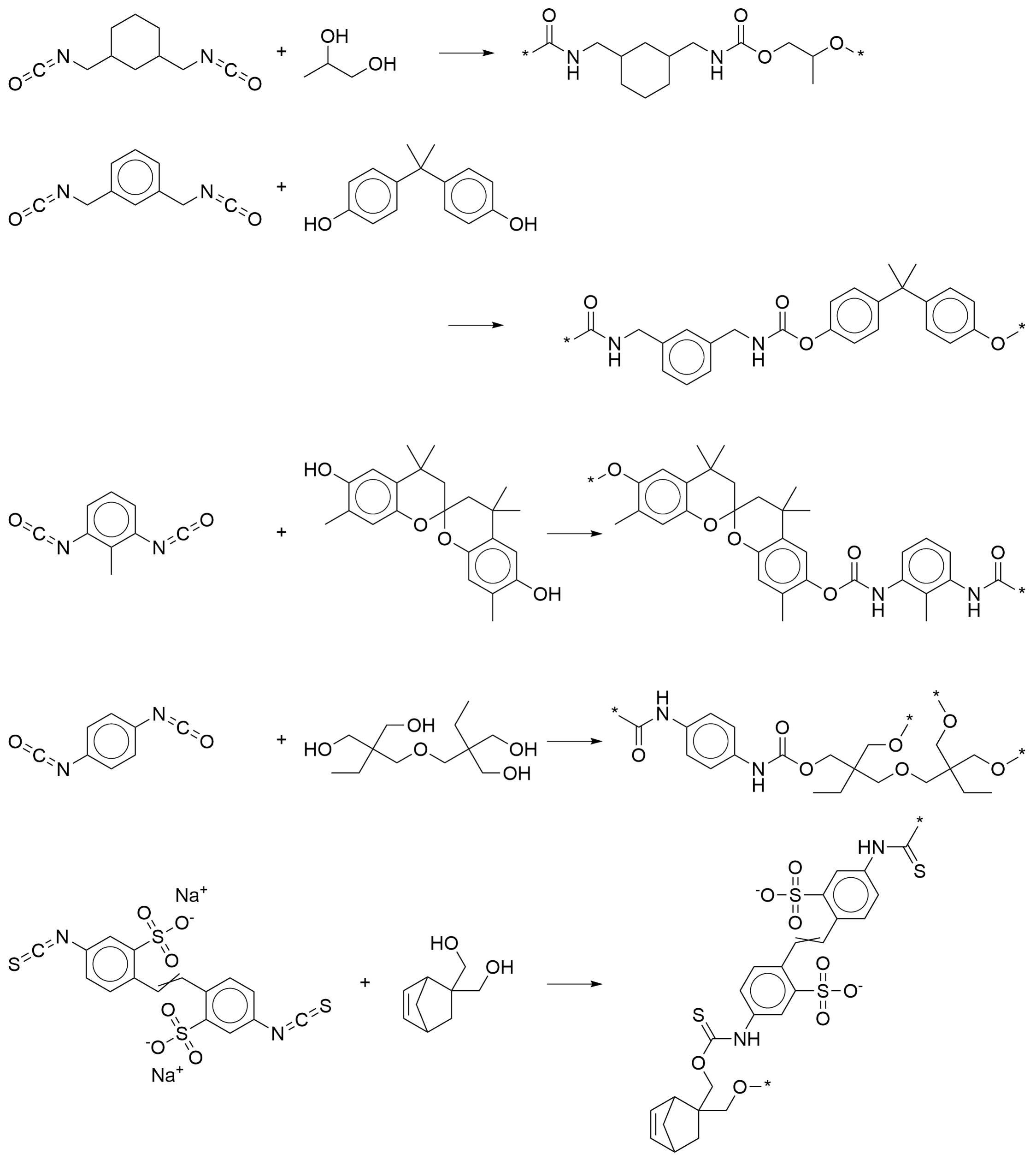

Figure S2: Examples of generated polymers belonging to seven different polymer classes and their polymerization reactions: (a) polyolefin, (b) polyester, (c) polyether, (d) polyamide, (f) polyimide and (g) polyoxazolidone *(continued)*.

(g) Polyoxazolidone (1/2)

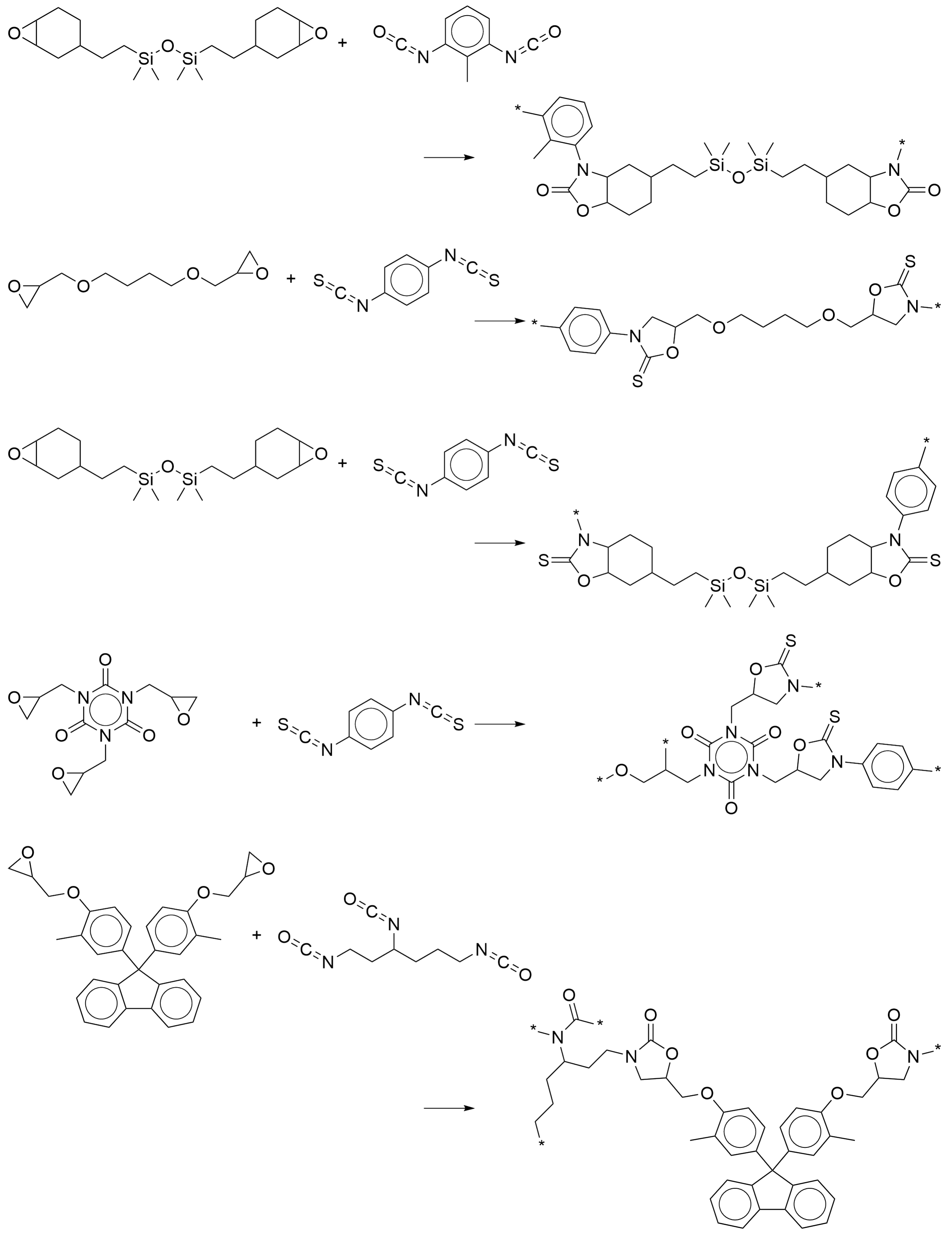

Figure S2: Examples of generated polymers belonging to seven different polymer classes and their polymerization reactions: (a) polyolefin, (b) polyester, (c) polyether, (d) polyamide, (f) polyimide and (g) polyoxazolidone *(continued)*.

(g) Polyoxazolidone (2/2)

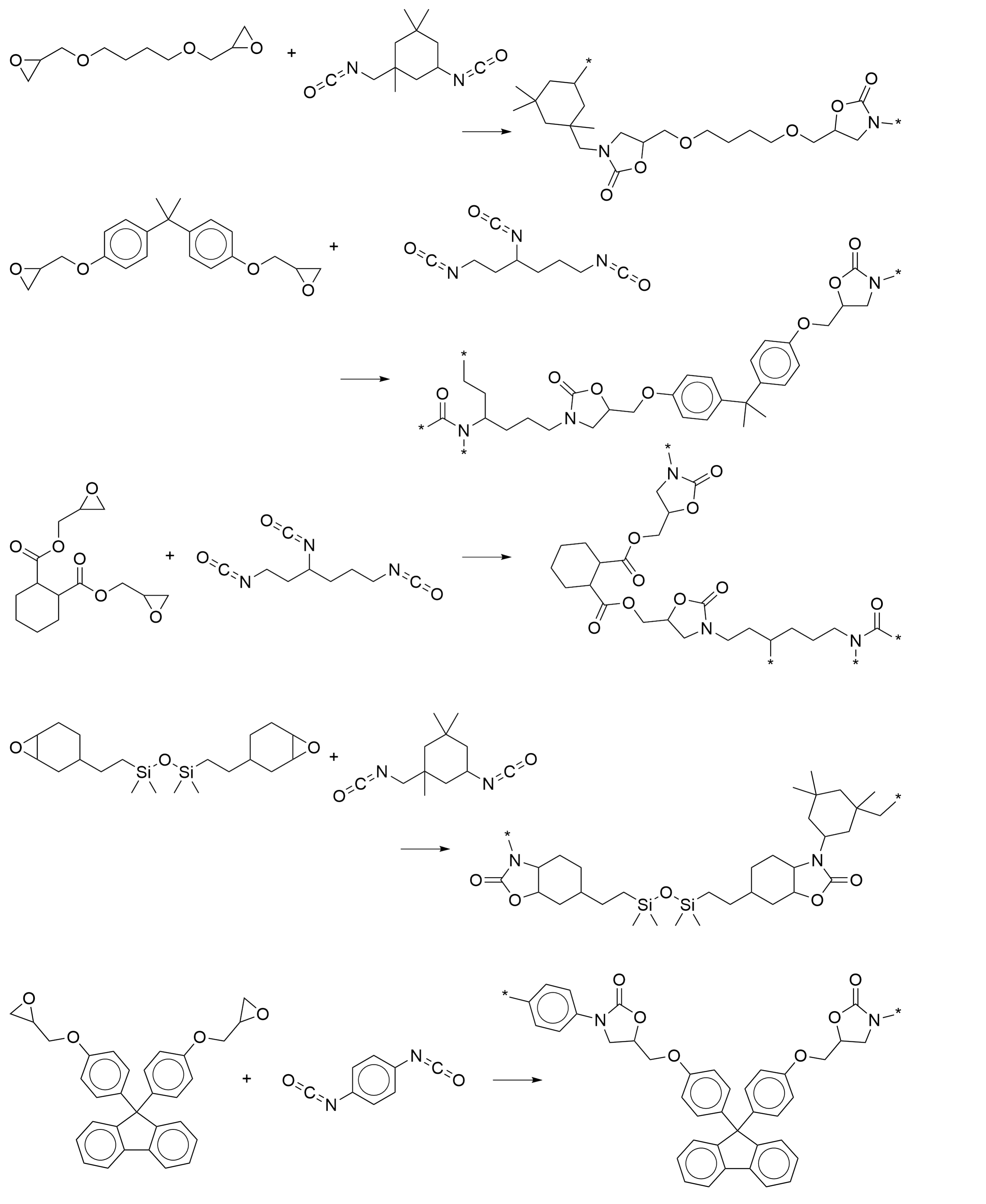

Figure S2: Examples of generated polymers belonging to seven different polymer classes and their polymerization reactions: (a) polyolefin, (b) polyester, (c) polyether, (d) polyamide, (f) polyimide and (g) polyoxazolidone *(continued)*.

4 Drawing coverage–novelty curve

As illustrated in Fig. S3, the CN curve shows an upward or downward convex pattern depending on the degree of overlap between the distribution of the virtual library and the existing polymers. When the two distributions coincide perfectly, the CN curve lies on a 45° line. If the virtual library cannot reproduce some of the existing polymers, the CN curve shows a downward convex pattern. If the virtual library covers the distribution of the existing polymers and generates a reasonable amount of novel structures, the CN curve shows a slightly upward convex pattern. Such a virtual library is most desirable.

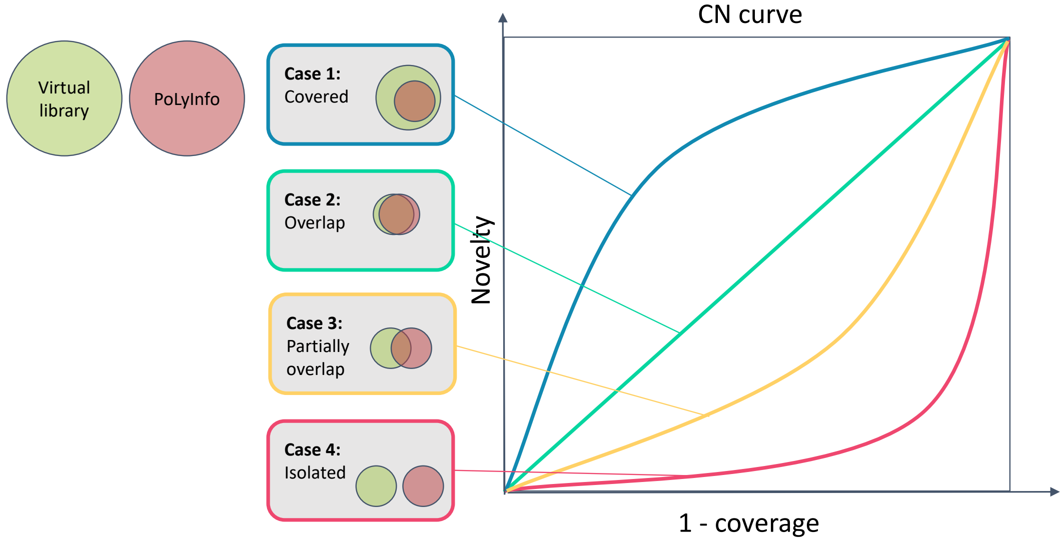

Figure S3: Change in the coverage–novelty curves due to the degree of overlap in the distribution of existing molecules and virtual polymers

5 Examples of polymers belonging to Regions A, B, and C in Figure 6

(a) Polymers belonging to Region A (1/2)

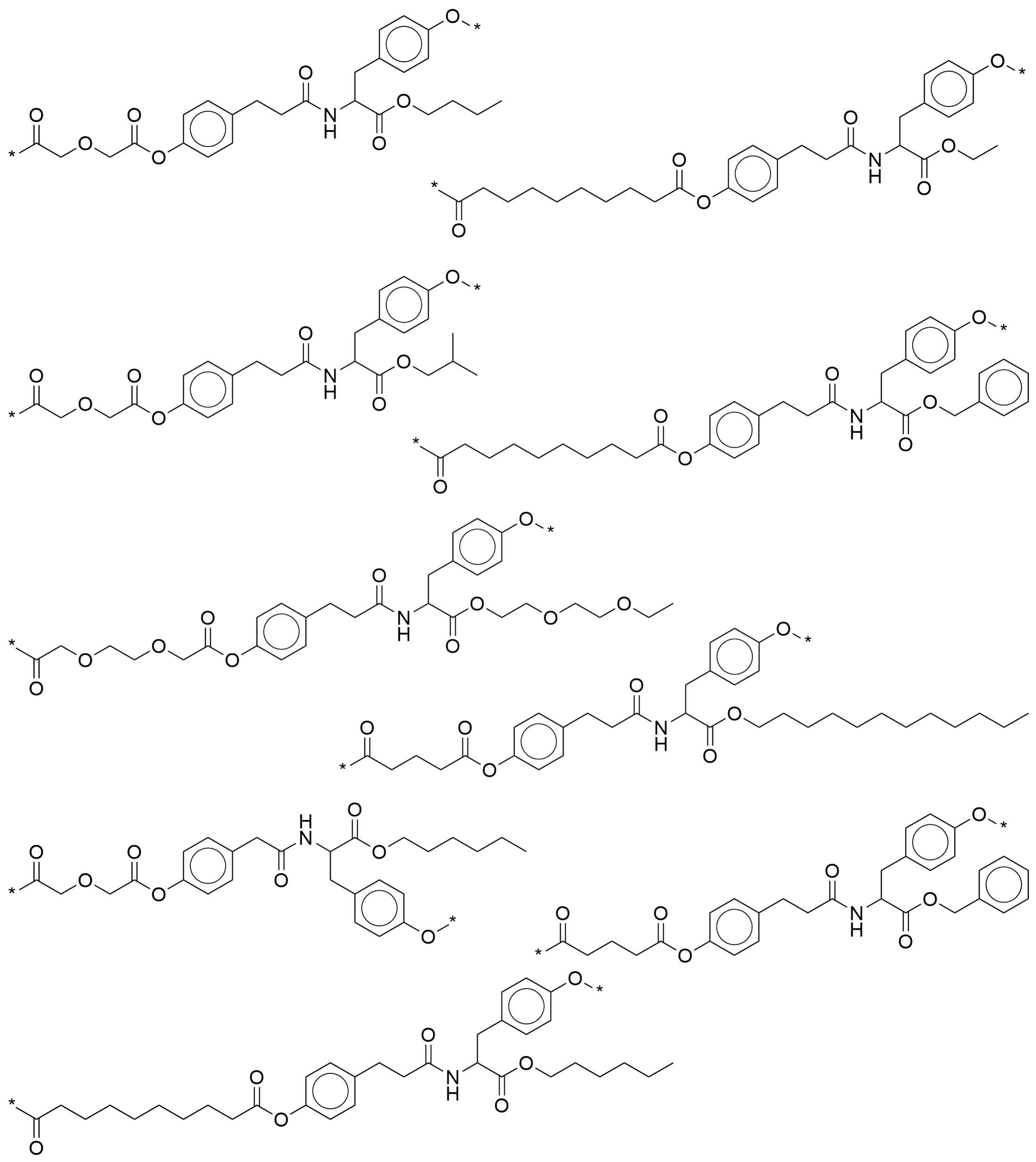

Figure S4: Polymers belonging to Region A, B and C in Figure 6 of the main text: (a) region A, (b) region B, (c) region C *(continued)*.

(a) Polymers belonging to Region A (2/2)

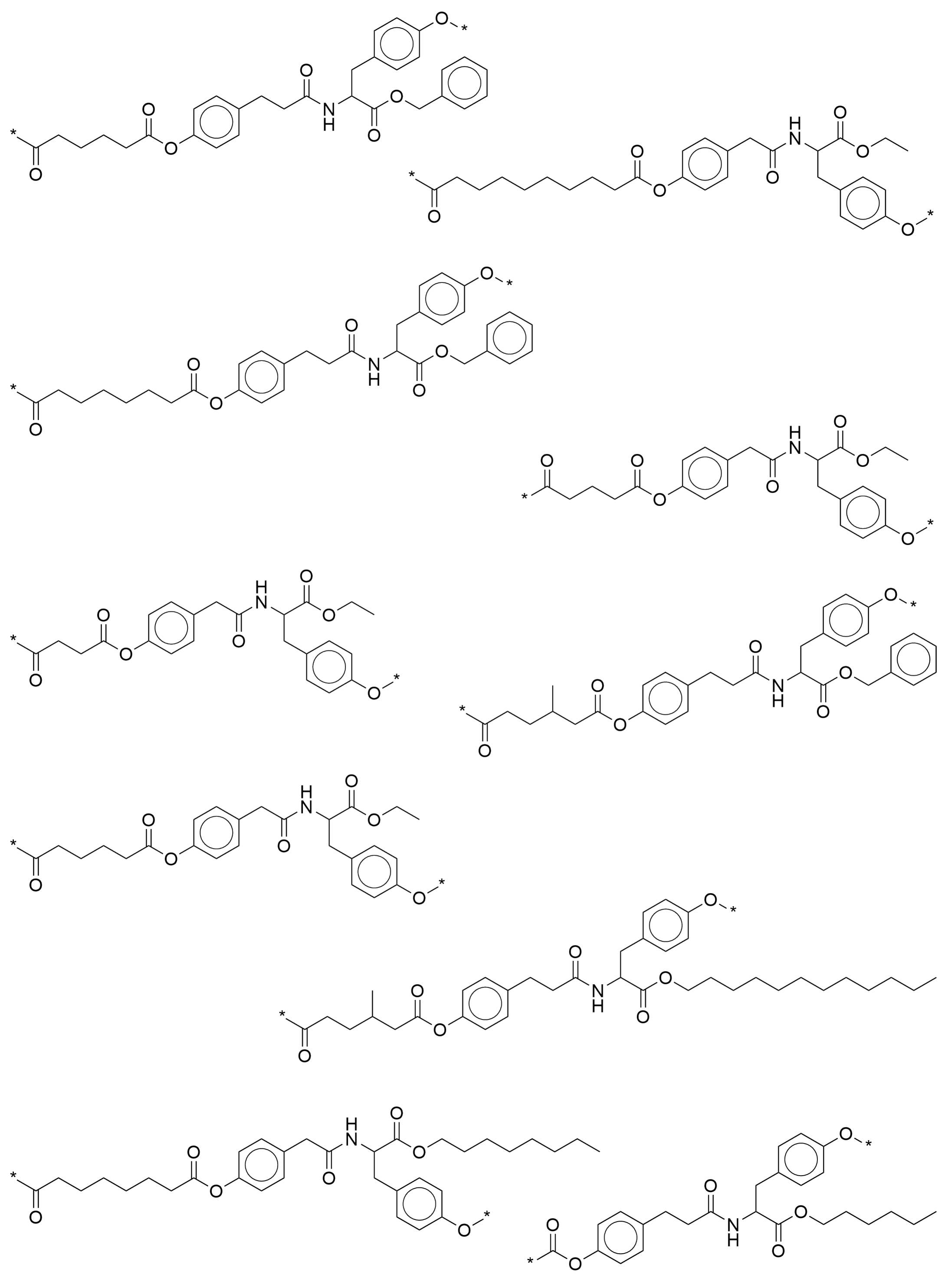

Figure S4: Polymers belonging to Region A, B and C in Figure 6 of the main text: (a) region A, (b) region B, (c) region C.

(b) Polymers belonging to Region B (1/2)

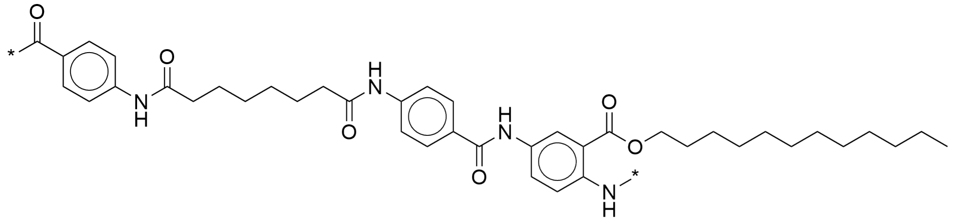

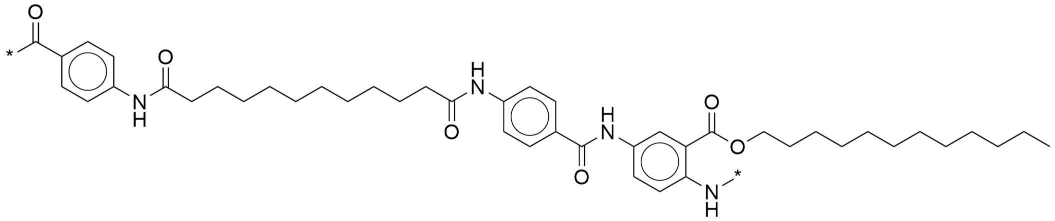

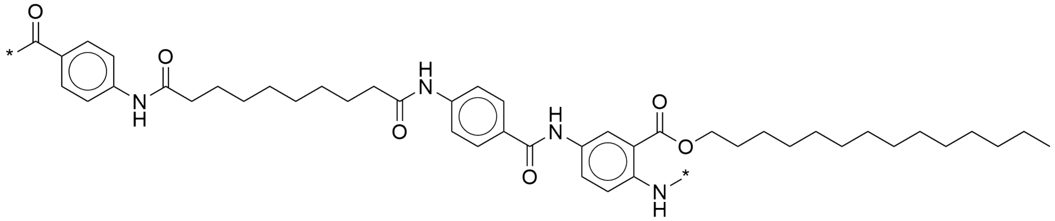

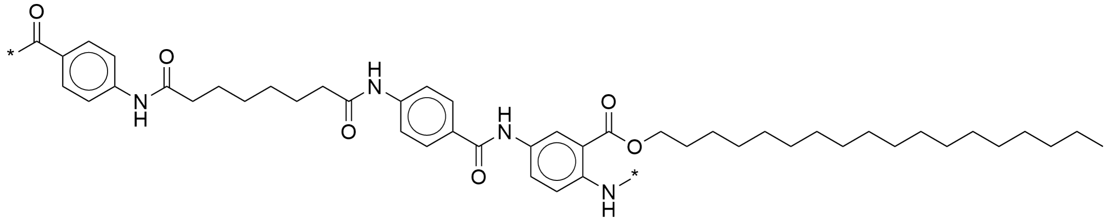

Figure S4: Polymers belonging to Region A, B and C in Figure 6 of the main text: (a) region A, (b) region B, (c) region C *(continued)*.

(b) Polymers belonging to Region B (2/2)

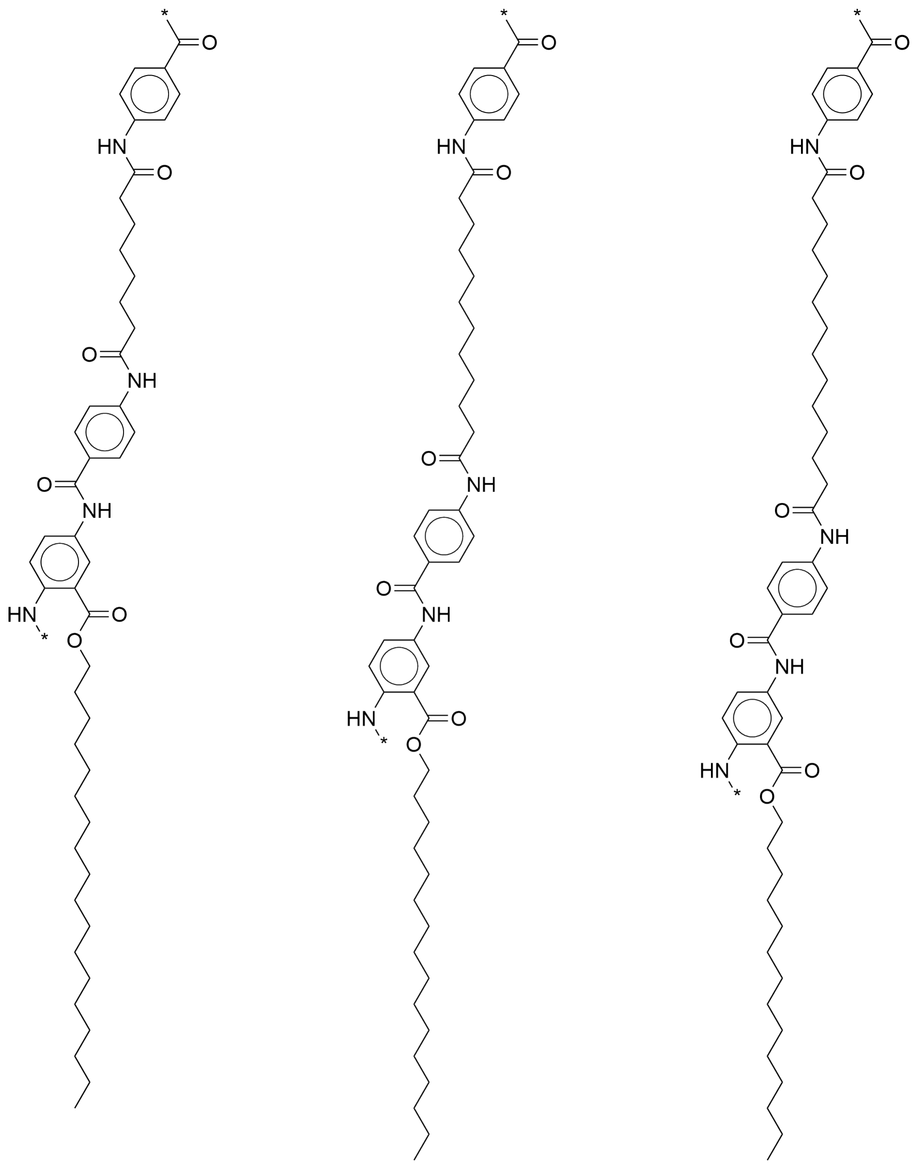

Figure S4: Polymers belonging to Region A, B and C in Figure 6 of the main text: (a) region A, (b) region B, (c) region C.

(c) Polymers belonging to Region C (1/3)

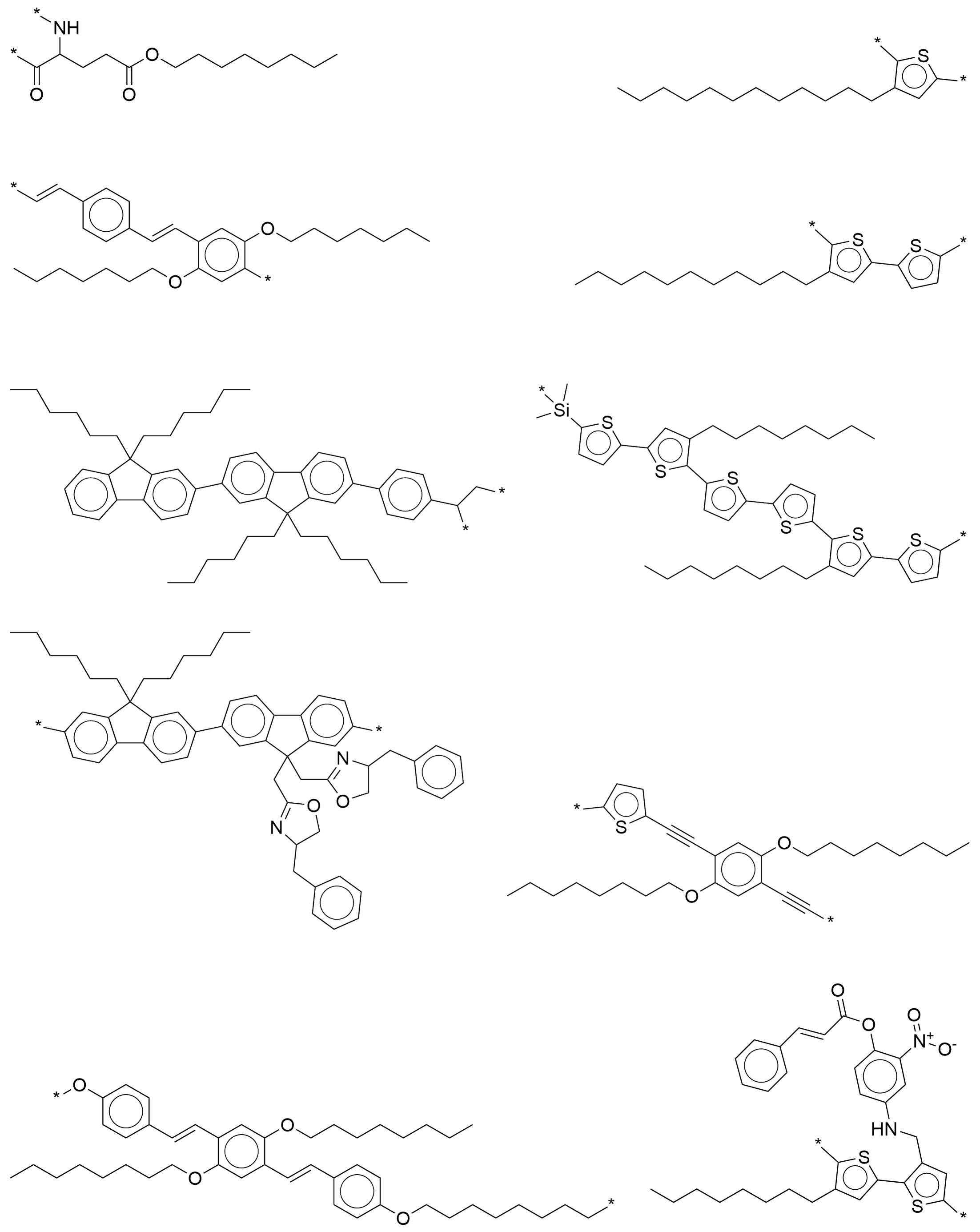

Figure S4: Polymers belonging to Region A, B and C in Figure 6 of the main text: (a) region A, (b) region B, (c) region C *(continued).*

(c) Polymers belonging to Region C (2/3)

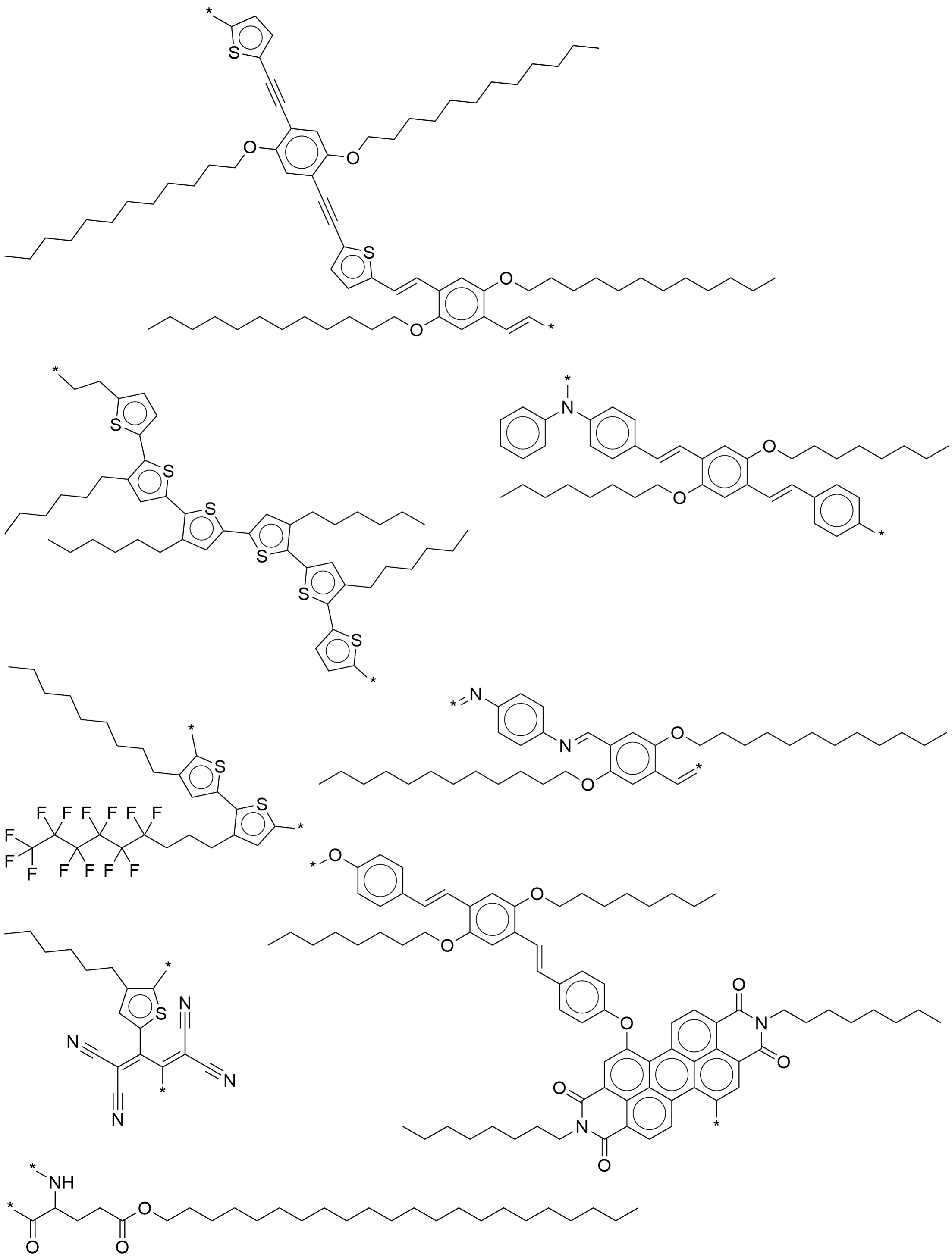

Figure S4: Polymers belonging to Region A, B and C in Figure 6 of the main text: (a) region A, (b) region B, (c) region C *(continued).*

(c) Polymers belonging to Region C (3/3)

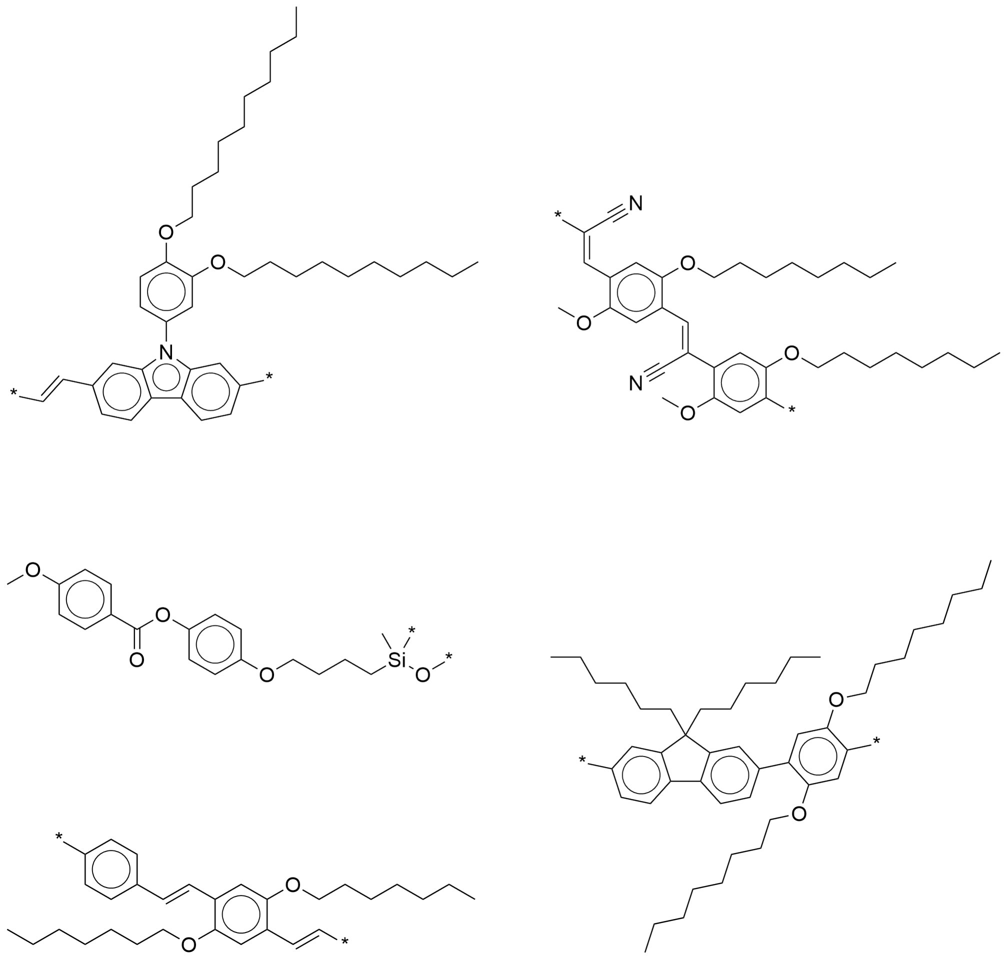

Figure S4: Polymers belonging to Region A, B and C in Figure 6 of the main text: (a) region A, (b) region B, (c) region C.

6 Comparison of polymerization reaction rule sets between SMiPoly and Open Macromolecular Genome

The correspondence between the polymerization reaction rules of SMiPoly and the Open Macromolecular Genome (OMG) is summarized in Table S2. The OMG contains approximately 12 million virtual polymers generated from 17 different polymerization reaction rules using approximately 80,000 small molecules as starting materials. Table S2 summarizes whether each of the 21 polymer classes of PoLyInfo can be generated by SMiPoly and OMG. Currently, SMiPoly implements 59% (10/17) of the reaction rules defined in the OMG, and the OMG implements 36% (8/22) of the reaction rules defined in SMiPoly.

Table S2: Correspondence of the polymerization reaction rules in SMiPoly and the OMG. The ability to generate for each of the PoLyInfo 21 polymer classes was compared.

polymer class		polymerization reaction	
PoLyInfo PID[a]	RadonPy	SMiPoly[b]	OMG[c]
P01, P31	hydrocarbon of polyolefin		
P02, P32	polystyrene	1	8
P03, P33	polyvinyl	3, 2, 4, 5	not defined
P04, P34	polyacrylate	(copolymer and cyclic olefin)	
P05, P35	halogenated polyolefin		
P06, P36	polydiene	not defined	9, 13, 16
P07, P37	polyether	12<br>13, 14, 15	12<br>not defined
P08, P38	polysulfide	not defined	15, 17
P09, P39	polyester	6<br>7<br>9<br>8, 11	10<br>7<br>3, 4<br>not defined
P10, P40	polyamide	16<br>19<br>17, 18	11<br>1, 2<br>not defined
P11, P41	polyurethane	21<br>22 (polyoxazolidone)	6<br>not defined
P12, P42	polyurea	not defined	5
P13, P43	polyimide	20	not defined
P14, P44	polyanhydride	not defined	not defined
P15, P45	polycarbonate	10<br>not defined	not defined<br>14
P16, P46	polyimine	not defined	not defined
P18, P48	polyphosphazene	not defined	not defined
P19, P49	polyketone	15 (PEK, etc.)	not defined
P20, P50	polysulfone	14 (PES, etc.)	not defined
P21, P51	polyphenylene	not defined	17

[a] The first three letters of PID in PoLyInfo.
[b] The reaction ID in Table 1 in the main text.
[c] The reaction ID in Figure 2 of Kim et al.[S1].

References

(S1) Kim, S.; Schroeder, C. M.; Jackson, N. E. Open Macromolecular Genome: Generative Design of Synthetically Accessible Polymers. *ACS Polymers Au* **2023**, in press, DOI: 10.1021/acspolymersau.3c00003.
